# Robust activation of microhomology-mediated end joining for precision gene editing applications

**DOI:** 10.1371/journal.pgen.1007652

**Published:** 2018-09-12

**Authors:** Hirotaka Ata, Thomas L. Ekstrom, Gabriel Martínez-Gálvez, Carla M. Mann, Alexey V. Dvornikov, Kyle J. Schaefbauer, Alvin C. Ma, Drena Dobbs, Karl J. Clark, Stephen C. Ekker

**Affiliations:** 1 Center for Clinical and Translational Science, Mayo Clinic, Rochester, MN, United States of America; 2 Graduate School of Biomedical Sciences, Mayo Clinic, Rochester, MN, United States of America; 3 Medical Scientist Training Program, Mayo Clinic, Rochester, MN, United States of America; 4 Genetics, Development, and Cell Biology, Iowa State University, Ames, IA, United States of America; 5 Biochemistry and Molecular Biology, Mayo Clinic, Rochester, MN, United States of America; 6 Department of Health Technology and Informatics, The Hong Kong Polytechnic University, Hong Kong, China; National Human Genome Research Institute (NIH), UNITED STATES

## Abstract

One key problem in precision genome editing is the unpredictable plurality of sequence outcomes at the site of targeted DNA double stranded breaks (DSBs). This is due to the typical activation of the versatile Non-homologous End Joining (NHEJ) pathway. Such unpredictability limits the utility of somatic gene editing for applications including gene therapy and functional genomics. For germline editing work, the accurate reproduction of the identical alleles using NHEJ is a labor intensive process. In this study, we propose Microhomology-mediated End Joining (MMEJ) as a viable solution for improving somatic sequence homogeneity *in vivo*, capable of generating a single predictable allele at high rates (56% ~ 86% of the entire mutant allele pool). Using a combined dataset from zebrafish (*Danio rerio*) *in vivo* and human HeLa cell *in vitro*, we identified specific contextual sequence determinants surrounding genomic DSBs for robust MMEJ pathway activation. We then applied our observation to prospectively design MMEJ-inducing sgRNAs against a variety of proof-of-principle genes and demonstrated high levels of mutant allele homogeneity. MMEJ-based DNA repair at these target loci successfully generated F0 mutant zebrafish embryos and larvae that faithfully recapitulated previously reported, recessive, loss-of-function phenotypes. We also tested the generalizability of our approach in cultured human cells. Finally, we provide a novel algorithm, MENTHU (http://genesculpt.org/menthu/), for improved and facile prediction of candidate MMEJ loci. We believe that this MMEJ-centric approach will have a broader impact on genome engineering and its applications. For example, whereas somatic mosaicism hinders efficient recreation of knockout mutant allele at base pair resolution via the standard NHEJ-based approach, we demonstrate that F0 founders transmitted the identical MMEJ allele of interest at high rates. Most importantly, the ability to directly dictate the reading frame of an endogenous target will have important implications for gene therapy applications in human genetic diseases.

## Author summary

New gene editing tools precisely break DNA at pre-defined genomic locations, but cells repair these lesions using diverse pathways that often lead to unpredictable outcomes in the resulting DNA sequences. This sequence diversity in gene editing outcomes represents an important obstacle to the application of this technology for human therapies. Using a vertebrate animal as a model system, we provide strong evidence that we can overcome this obstacle by selectively directing DNA repair of double-stranded breaks through a lesser-described pathway termed Microhomology-mediated End Joining (MMEJ). Unlike other, better-understood pathways, MMEJ uses recurring short sequence patterns surrounding the site of DNA breakage. This enables the prediction of repair outcomes with improved accuracy. Importantly, we also show that preferential activation of MMEJ is compatible with effective gene editing. Finally, we provide a simple algorithm and software for designing DNA-breaking reagents that have high chance of activating the MMEJ pathway. We believe that the MMEJ-centric approach to be broadly applicable for a variety of gene editing applications both within the laboratory and for human therapies.

## Introduction

**Fig 1:**
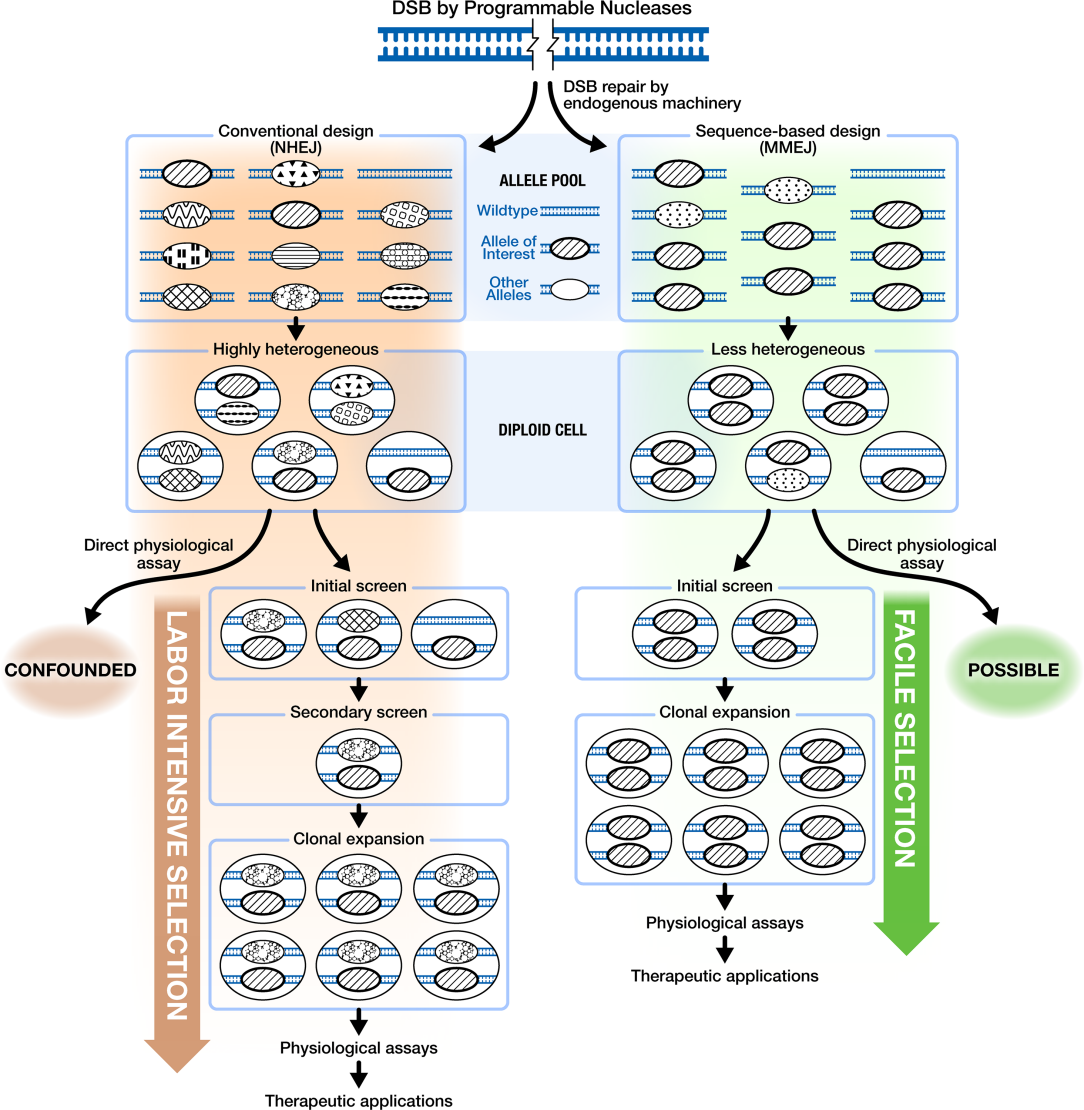
MMEJ is a unique DSB repair pathway that results in highly efficient and highly stereotyped mutagenesis. DSB by conventionally designed Programmable Nucleases typically proceeds through a versatile yet unpredictable classical non-homologous end joining (NHEJ) pathway. As a result, a rather diverse cohort of mutant alleles are generated, making the subsequent selection process labor intensive to enrich for the allele of interest. The resulting genetic composition of the specific loci are often complex, requiring careful molecular characterization of each allele. Efficient activation of microhomology-mediated end joining (MMEJ) pathway, on the other hand, can greatly limit allelic diversity and enable the intentional generation of a particular deletion allele of interest at a high rate. Consequently, the downstream applications become more streamlined with facile generation of homozygous frameshift allele in diploid cells.

Programmable nucleases such as TALEN (Transcription Activator-like Effector Nuclease) and CRISPR (Clustered Regularly Interspaced Short Palindromic Repeats) systems have enabled a new era of scientific research [1, 2]. Instead of relying on knock-down models or expensively outsourced knock out lines, laboratories across the world now have tools with which to generate indels (insertions and deletions) of varying sizes on the gene(s) of interest. However, DNA Double-strand Break (DSB) repairs largely result in diverse sequence outcomes owing to the unpredictable nature of the most commonly used Non-homologous End Joining (NHEJ) pathway [3, 4] (**Fig 1**). This significantly confounds experimental readouts as knock-out cell lines often harbor more than just one desired frameshift mutation. In the case of model organisms such as zebrafish (*Danio rerio*), the F0 founders are genetically mosaic, warranting a complex and time-consuming series of outcrossing to establish molecularly defined lines before any biological questions can be addressed [5, 6].

In contrast to NHEJ, the MMEJ (Microhomology-mediated End Joining) DNA repair pathway utilizes a pair of locally available direct sequence repeats on both sides of a DSB that are apposed, annealed and extended [7–10]. As such, DSB repair outcomes are highly stereotyped (**Fig 1**), resulting in deletion of the intervening sequence as well as one of the repeats. Consequentially, there is an increasing interest in utilizing MMEJ for precision genome engineering applications [11–14]. To date, however, effective harnessing of this pathway remains challenging due to the paucity of genetic and mechanistic understanding [8].

Bae, *et al*. [14] developed a sequence-based scoring system to estimate the frequency of MMEJ-associated deletions induced by DSBs in human cells. While this improved the predictability of MMEJ activation, the DSB repair outcomes tended to consist of a heterogeneous population of multiple MMEJ alleles. In this study, we sought to improve upon the existing algorithm with the goal of developing tools to more reliably predict target loci that would be predisposed to generate a more homogeneous mutant allele population through MMEJ. We demonstrate the feasibility and utility of such reagent design on the molecular level (i.e., DNA repair outcomes) and on the physiological level (i.e., F0 phenotype). We further demonstrate that our approach can be applied to generating highly homogeneous MMEJ alleles in cultured human cells, suggesting our findings may be broadly translatable to multiple model systems. We believe our approach can inform and benefit applications such as rapid phenotype-genotype correlation in F0 animals, with an eye toward applications in human gene therapy and facilitation of resource sharing & recreation of various cell and animal lines on a global scale.

## Results

### MMEJ is an active repair pathway in the genetically unaltered zebrafish embryo

**Fig 2:**
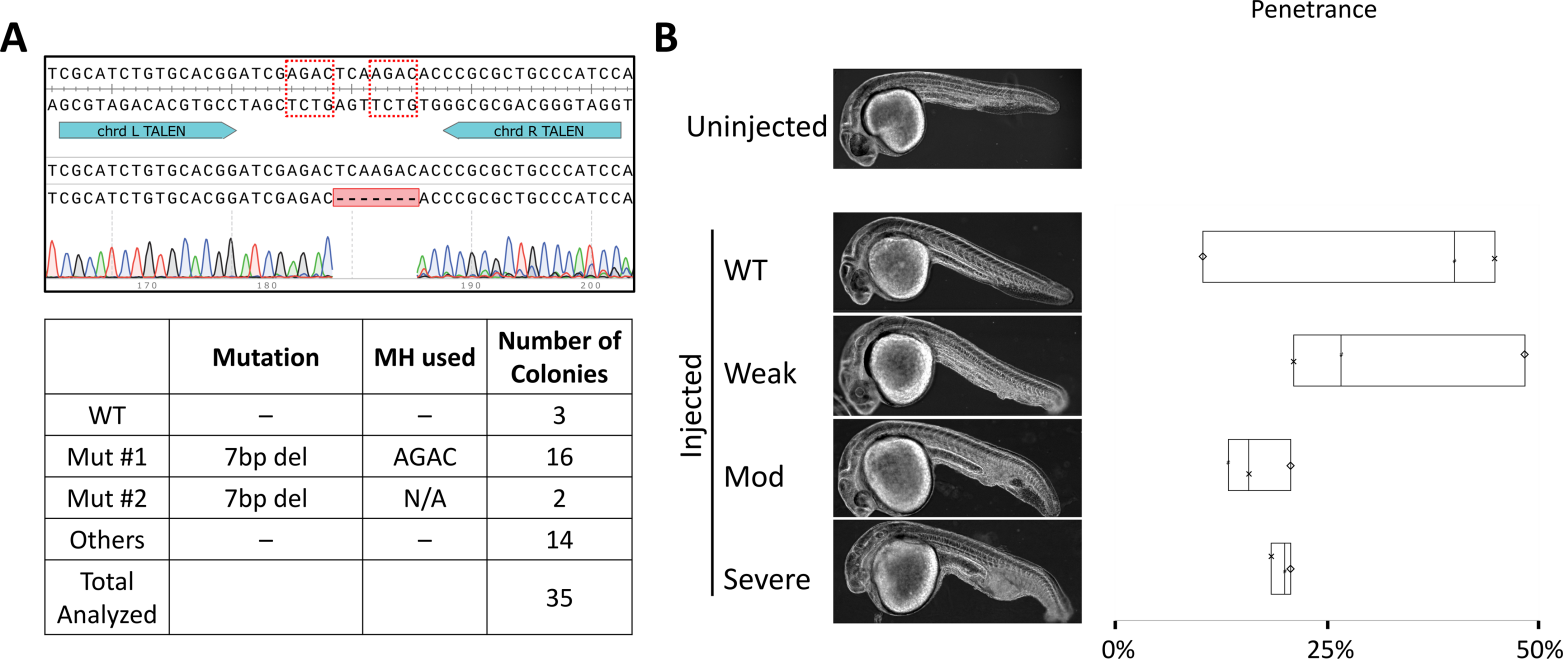
PreMA TALEN reagent can be used to recapitulate previously reported loss-of-*chrd*-function phenotype in 1 dpf F0, injected larvae. **A.** *Top–*Wildtype *chrd* sequence with TALEN binding sites annotated in teal. The dotted red boxes are MH arms predicted to be used most frequently. Raw sequence alignment of the whole PCR amplicon demonstrates that the majority of reads are the expected 7 bp deletion allele. *Bottom*–summary data from subcloning analyses. 50% of the mutant allele recovered were of the predicted MH allele. **B.** Previously reported *chrd* loss-of-function phenotype was successfully recapitulated using this TALEN pair. Phenotype severity was graded by the degree of Intermediate-Cell-Mass expansion in the tail and by the reduced head size by 1 dpf. Box plot demonstrating phenotypic penetrance is provided with each experiment denoted by a unique marker shape. N = 3 biological and technical replicates. At least 29 injected animals were scored in each experiment.

**Fig 3:**
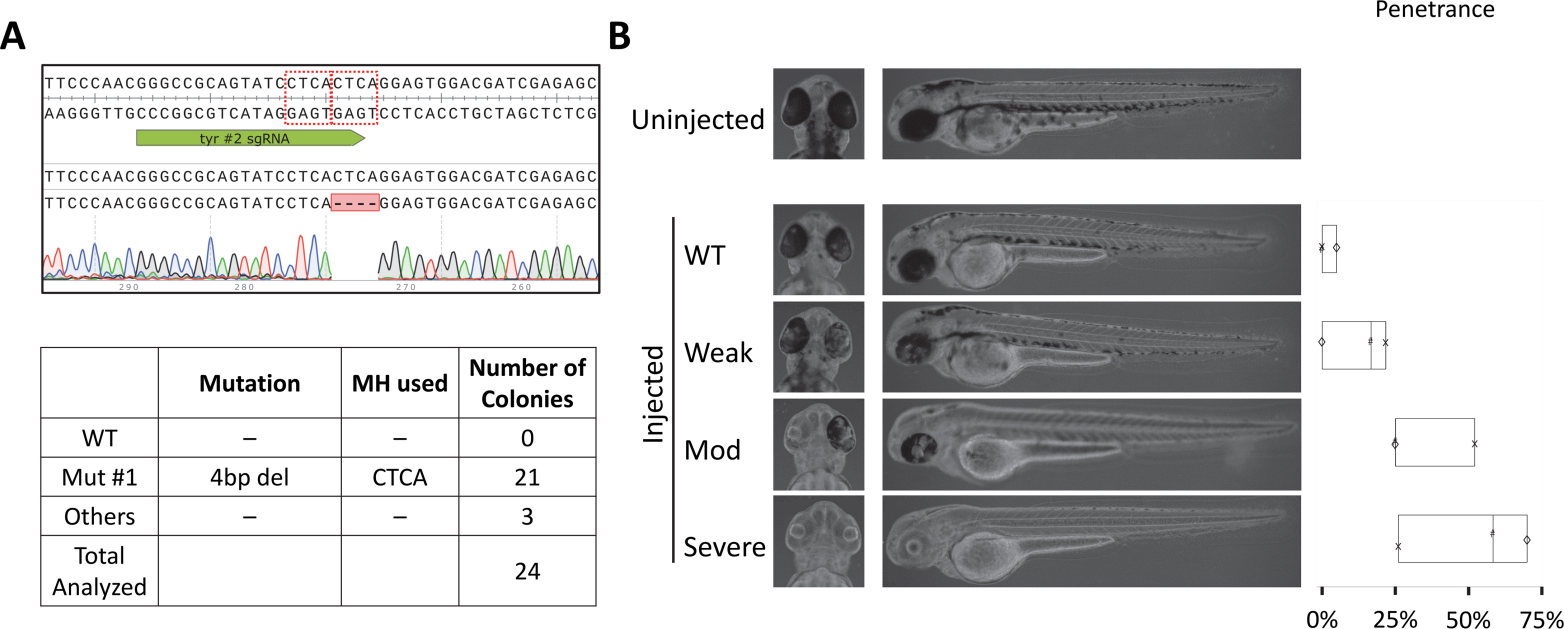
PreMA sgRNA against *tyr* can be used to recapitulate loss-of-melanophore phenotype in 2 dpf, injected F0 larvae. **A.** *Top–*Wildtype *tyr* sequence with the #2 sgRNA target site annotated in green. The dotted red boxes are MH arms predicted to be used most frequently. Raw sequence alignment of the whole PCR amplicon demonstrates that the majority of reads are the expected 4 bp deletion allele. *Bottom*–summary data from subcloning analyses. 88% of the mutant allele recovered were of the predicted MH allele. **B.** Previously reported *tyr* loss-of-function phenotype was successfully recapitulated using this CRISPR-Cas9. Phenotype severity was graded by the loss of retinal pigmentation. Partial loss of retinal pigmentation was considered a Weak phenotype, whereas complete loss of pigmentation in one or both eyes were considered Moderate and Severe phenotypes, respectively. Box plot demonstrating phenotypic penetrance is provided with each experiment denoted by a unique marker shape. N = 3 biological and technical replicates. At least 12 injected animals were scored in each experiment.

Prior works examining MMEJ activation in vertebrate organisms primarily focused on *in vitro* models [8–10, 14–18]. Initial analyses using a targeted knockin strategy suggested that MMEJ was operational in the zebrafish embryo, though the efficiency of these MMEJ outcomes was rather modest [13]. Importantly, while previous studies reported incidental identification of several zebrafish genomic loci that repaired preferentially through MMEJ when using programmable nucleases [19, 20], no consortium–small or large–of genomic loci that repair primarily through NHEJ vs MMEJ has been compiled. To this end, we examined the repair outcomes of previously designed TALEN and CRISPR-Cas9 genomic reagents (**S1 Table**). The plurality of custom enzymes induced diverse sequence outcomes, consistent with the idea that NHEJ is being used as the primary DNA repair pathway at these loci. However, a few reagents induced sequence outcomes satisfying the following criteria, suggesting that MMEJ was the preferred pathway: 1) most predominant mutant allele is the top predicted allele by the Bae, *et al* algorithm [14], 2) most predominant mutant allele comprises ≥ 50% of the total mutant allele population, and 3) mutagenic efficiency > 20%. For the purpose of this study, a programmable nuclease satisfying all these criteria is referred to as a Predominant MMEJ Allele (PreMA) reagent. Three sticky-end generating TALEN (*chrd*, *mitfa* #4 & *surf1*) and two blunt-end generating CRISPR-Cas9 (*surf1* & *tyr* #2) reagents fell into this category (**S1 Table**, **Fig 2A**, **Fig 3A**).

Injecting the *chrd* TALEN pair (37.5 pg/arm) resulted in characteristic *chrd* loss of function phenotypes: Intermediate-Cell-Mass expansion and a smaller head by 1 day post-fertilization [21] (1 dpf; **Fig 2B**). Median penetrance for Moderate and Severe phenotypes was 15.8% and 20.0%, respectively (**Fig 2B**, **S2 Table**). Strong MMEJ activation by this TALEN pair was confirmed by subcloning analysis (**Fig 2A**)– 16/32 recovered mutant reads corresponded to the top predicted 7 bp deletion allele. Similarly, perturbing *tyr* gene with a CRISPR-Cas9 reagent recapitulated a previously reported, loss of melanin production phenotype, observable by 2 dpf [22] (**Fig 3B**). Ribonucleoprotein (RNP) delivery at the dose of 300 pg *tyr* #2 sgRNA and 660 pg Cas9 resulted in Moderate and Severe loss of pigmentation phenotypes in 22.7% and 50.0% of embryos respectively (**Fig 3B**, **S2 Table**). Subcloning analysis showed 21/24 (88%; **Fig 3A**) of resulting alleles contained a 4 bp deletion consistent with a strong MMEJ activation by this CRISPR-Cas9. Together with the *chrd* TALEN results, these data support that MMEJ can be an effective repair pathway in F0 embryos at some genomic loci, irrespective of programmable nucleases used.

### Many Bae, *et al*. predicted MMEJ loci are preferentially repaired by NHEJ

A subset of these zebrafish reagents described above was prospectively designed using the Bae, *et al*. algorithm (**S1 Table**). This algorithm calculates the strength of each pair of microhomology arms (i.e., *Pattern Score*) according to the length and GC content of each pair, as well as the length of the intervening sequence. The additive sum of all the possible *Pattern Scores* is then returned as *Microhomology Score*. This latter score was found to have positive correlation with the rate of MMEJ activation in HeLa cells [14]. All fourteen prospectively designed reagents had a *Microhomology Score* of at least 4000 –a median score found on human *BRCA1* gene. However, only four of these reagents induced majority MMEJ outcomes as judged by the *Microhomology Fraction* (**S1 Table**, **S1 Note**). We therefore retrospectively analyzed the repair outcomes of these reagents to identify additional factor(s) that would enhance predictability of MMEJ induction.

### Rate of *Pattern Score* change as a discrimination factor for MMEJ induction *in vivo* and *in vitro*

Intriguingly, when the pattern score values clustered closely to one another (i.e., a flatter *Slope Value* as calculated according to **S2 Note**), this was indicative of an unfavorable target for MMEJ activation in zebrafish embryos. Conversely, loci at which *Pattern Scores* dropped precipitously (i.e., a steeper *Slope Value*) were good candidates of MMEJ activation *in vivo* (p = 0.0048; **S1 Fig**). Based on these observations, we hypothesized that locally available microhomology pairs are in direct competition with one another such that overabundance of these pairs is a negative predictor of MMEJ activation. In other words, MMEJ activation is more favorable at loci with one or two predominant microhomology pair(s) (*Low Competition* loci) rather than many strong microhomology pairs (*High Competition* loci).

To determine whether the zebrafish-based hypothesis was generalizable to human cells (HeLa), we re-analyzed the deep sequencing dataset used to generate the Bae, *et al*. algorithm [14]. Available results from 90 genomic loci were sorted alphabetically by the names of target genes then divided into two groups: first 50 and the remaining 40. The first group was then used for a retrospective, correlative analysis while the latter was used for an analysis compatible with a prospective study design. Outcomes from the first 50 targets showed a correlation similar to that observed in zebrafish; higher *Microhomology Fractions* generally correlated with low *Slope Values* (p = 0.00001; **S2A Fig**). This correlation was lost when microhomology arms of 2 bp were included in the analysis (p = 0.2644; **S2B Fig**); accordingly, microhomology arms of less than 3 bp were excluded from subsequent analyses. The remaining 40 targets were then binned into *High*, *Medium* and *Low Competition* groups based on quartile distribution of the *Slope Value* (**S2C Fig**). In agreement with our Competition Hypothesis, the median *Microhomology Fraction* was significantly higher in the *Low Competition* group than in the *High Competition* group (0.300 vs 0.105, p = 0.011; **S2D Fig**).

### Competition hypothesis predicts new PreMA reagents

Based on this Competition Hypothesis, we designed 20 *Low Competition* sgRNA targets across 9 genes and analyzed the DSB repair outcomes (**S3 Table**). *Slope Values* smaller than -40 was used as the cut-off for Low Competition, as 3 out of 4 previously designed zebrafish targets produced majority MMEJ outcomes in this range (**S1 Table and S1 Fig**). For initial assessments, we used TIDE (Tracking Indels by DEcomposition) analysis–a chromatogram analyzing tool that estimates proportions of length varying mutant alleles present in a pool of mixed alleles [23]–which revealed that 5 of these sgRNAs against 3 genes (*mtg1*, *tdgf1*, *ttn*.*2 #1*, *ttn*.*2 #2*, and *ttn*.*2* N2B #1) were in the PreMA class. These results were subsequently confirmed by subcloning analyses (**S3 Table**).

**Fig 4:**
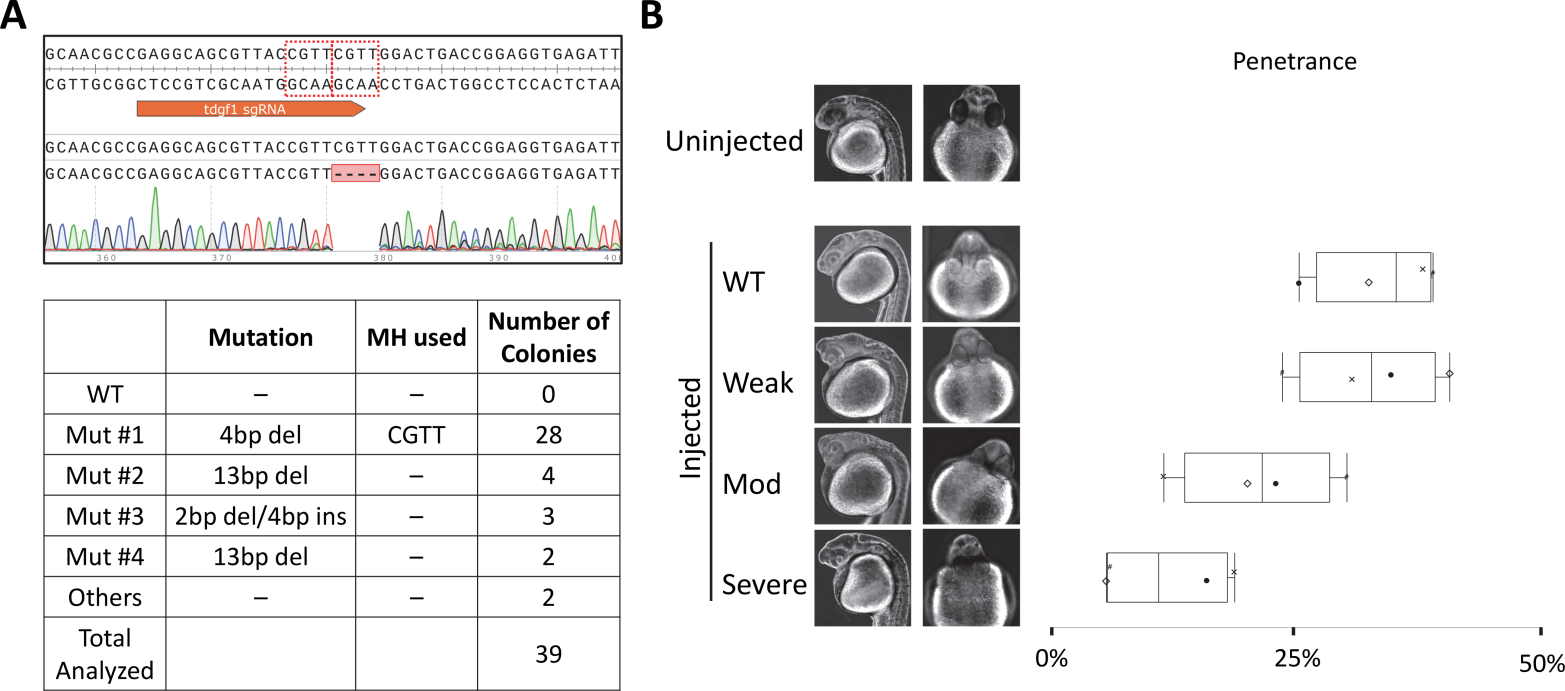
Prospectively designed PreMA reagent against *tdgf1* can be used to reproduce gross developmental defect in 1 dpf, injected F0 larvae. **A.** *Top–*Wildtype *tdgf1* sequence with sgRNA target site annotated in orange. The dotted red boxes are MH arms predicted to be used most frequently. Raw sequence alignment of the whole PCR amplicon demonstrates that the majority of reads are the expected 4 bp deletion allele. *Bottom*–summary data from subcloning analyses. 72% of the mutant allele recovered were of the predicted MH allele. **B.** Previously reported *tdgf1* loss-of-function phenotype was successfully recapitulated using this CRISPR-Cas9. Phenotype severity was graded by the “pinhead” morphology and cyclopia. Pinhead morphology alone was classified as Weak, whereas Moderate and Severe phenotypes also presented with varying degrees of cyclopia judged by the distance of forebrain protrusion. In the Severe class, the forebrain does not separate the eyes, and they are fused together. Box plot demonstrating phenotypic penetrance is provided with each experiment denoted by a unique marker shape. N = 4 with 3 biological and 4 technical replicates. At least 42 injected animals were scored in each experiment.

Perturbation of *tdgf1* (alternatively known as *One-eyed Pinhead*) causes aberrant, “pinhead” morphology and cyclopia as judged by reduced forebrain protrusion by 1 dpf [24] (**Fig 4B**). RNP injections of CRISPR-Cas9 at the dose of 300 pg sgRNA and 660 pg Cas9 resulted in highly homogeneous DSB repair outcomes, generating the top-predicted 4bp allele in 28 of 39 clones analyzed (**Fig 4A**). Aberrant head morphology alone was classified as Weak whereas that in combination with varying degrees of forebrain protrusion was classified as Moderate or Severe phenotypes. Median penetrance for Moderate and Severe morphology was 21.8% and 11.4% (**Fig 4B**, **S2 Table**), consistent with the subcloning results.

**Fig 5:**
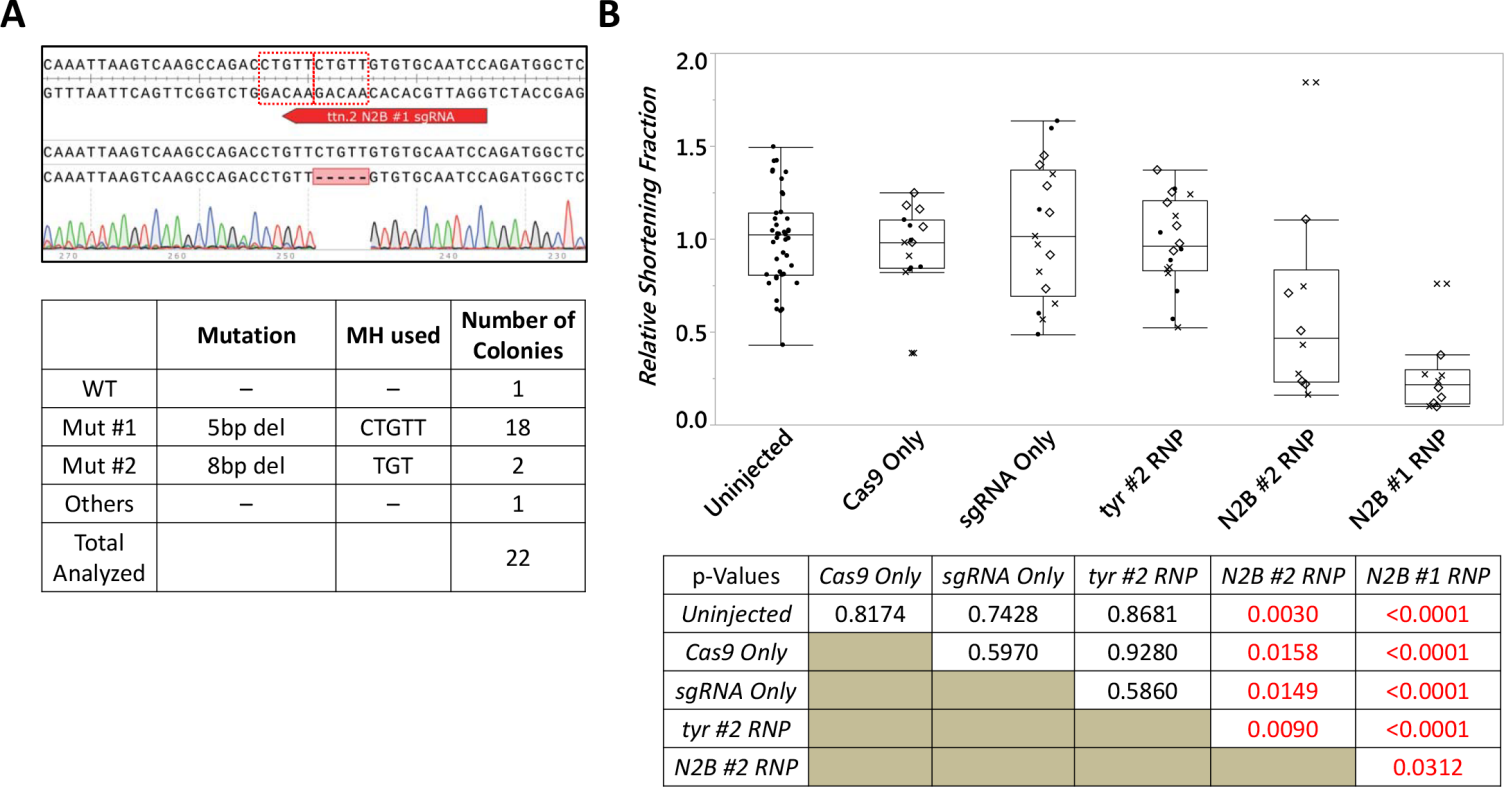
PreMA reagent against *ttn*.*2* N2B results in specific reduction of shortening fraction in 2 dpf F0 zebrafish. **A.** *Top–*Wildtype *ttn*.*2* sequence at the N2B exon with sgRNA target site annotated in red. The dotted red boxes are MH arms predicted to be used most frequently. Raw sequence alignment of the whole PCR amplicon demonstrates that the majority of reads are the expected 5 bp deletion allele. *Bottom*–summary data from subcloning analyses. 86% of the mutant allele recovered were of the predicted MH allele. **B.** Previously reported *pickwick* phenotype was successfully recapitulated using this CRISPR-Cas9. 2 dpf zebrafish were immobilized in 3% methylcellulose for live recording of cardiac functions. Whereas injections with Cas9 only (660 pg), N2B #1 sgRNA only (300 pg), or *tyr* #2 sgRNA RNP (300 pg sgRNA + 660 pg Cas9) did not result in changes in shortening fraction at this age, MMEJ-inducing RNP injection targeting N2B #1 (300 pg sgRNA + 660 pg Cas9) resulted in a specific reduction in shortening fraction by 78.4%. In contrast, NHEJ-inducing RNP injection targeting N2B #2 (300 pg sgRNA + 660 pg Cas9) resulted in attenuated effects on shortening fraction (53.3% reduction), despite similarly high edit efficiency. Each data point represents an individual animal scored with the shape of the marker denoting unique experiment. N ≥ 3 biological and technical replicates, except for N2B #2 where N = 2. At least 5 injected animals were scored in each experiment. P-values calculated by Wilcoxon’s Each Pair Calculation (adjusted for multiple comparisons).

We next explored whether these PreMA reagents are useful for recapitulating a more subtle phenotype beyond aberrant gross morphologies observed in the *tdgf1* mutants. Splice blockade at the N2B exon of *ttn*.*2* gene by a synthetic morpholino oligonucleotide was previously reported to reduce the cardiac contractility by ~70% on 2 dpf [25], phenocopying the *pickwick*^m171^ mutation [26]. RNP delivery at the dose of 300 pg *ttn*.*2* N2B #1 sgRNA + 660 pg Cas9 resulted in reduction of the shortening fraction to a comparable degree (**Fig 5B**). Importantly, RNP delivery of NHEJ-inducing *ttn*.*2* N2B #2 sgRNA at the same dose only resulted in a more attenuated phenotype, despite it targeting the same exon and having comparable activity (**Fig 5; S4 Table**). Due to the high editing efficiency, animals injected with these doses of *ttn*.*2* N2B #1 RNP were not viable in post larval phases. For this reason, animals injected at the lower dose of 75 pg sgRNA + 165 pg Cas9 protein were raised to adulthood. Two F0 founders were successfully outcrossed to wildtype zebrafish. Heterozygous offspring were identified using the dsDNA heteroduplex-cleaving Surveyor assay [27], and the transmission of the top predicted 5 bp deletion allele was confirmed from both founders by subcloning analyses (**S3 Fig**).

**Fig 6:**
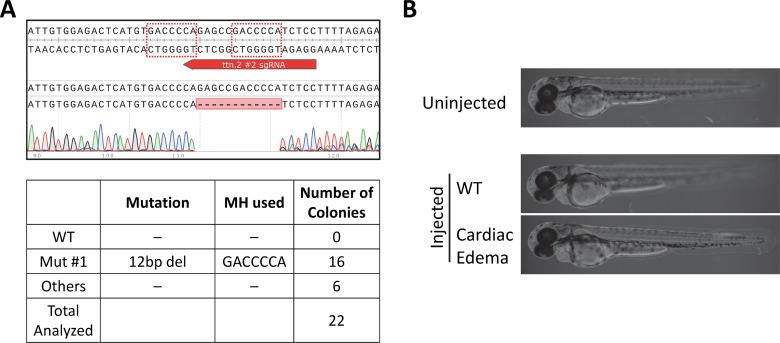
PreMA reagent can be used for in-frame gene alteration. **A.** *Top–*Wildtype *ttn*.*2* sequence with sgRNA target site annotated in red. The dotted red boxes are MH arms predicted to be used most frequently. Raw sequence alignment of the whole PCR amplicon demonstrates that the majority of reads are the expected 12 bp deletion allele. *Bottom*–summary data from subcloning analyses. 73% of the mutant allele recovered were of the predicted MH allele. **B.** 2 dpf zebrafish larvae injected with *ttn*.*2 #2* sgRNA RNP (300 pg sgRNA + 660 pg Cas9) grossly appear normal with the exception of mild cardiac edema. Median penetrance was 50%. N = 3 biological and technical replicates. At least 9 injected animals were scored in each experiment.

We also designed an sgRNA against exon 13 of *ttn*.*2* (*ttn*.*2* #2 sgRNA), expected to produce a 12 bp deletion allele as a proof-of-principle for in-frame gene correction (**Fig 6A**). RNP delivery at the dose of 300 pg sgRNA + 660 pg Cas9 resulted in the induction of this 12 bp deletion allele in 72.7% of the clones. While the injected animals presented with mild cardiac edema evident by 2 dpf (median rate: 50.0%; **Fig 6B**, **S2 Table**), unlike the N2B #1 sgRNA CRISPR-Cas9 injected animals, these were viable to adult age.

### Low competition plus proximity of microhomology arms strongly predicts PreMA reagents: V2

**Fig 7:**
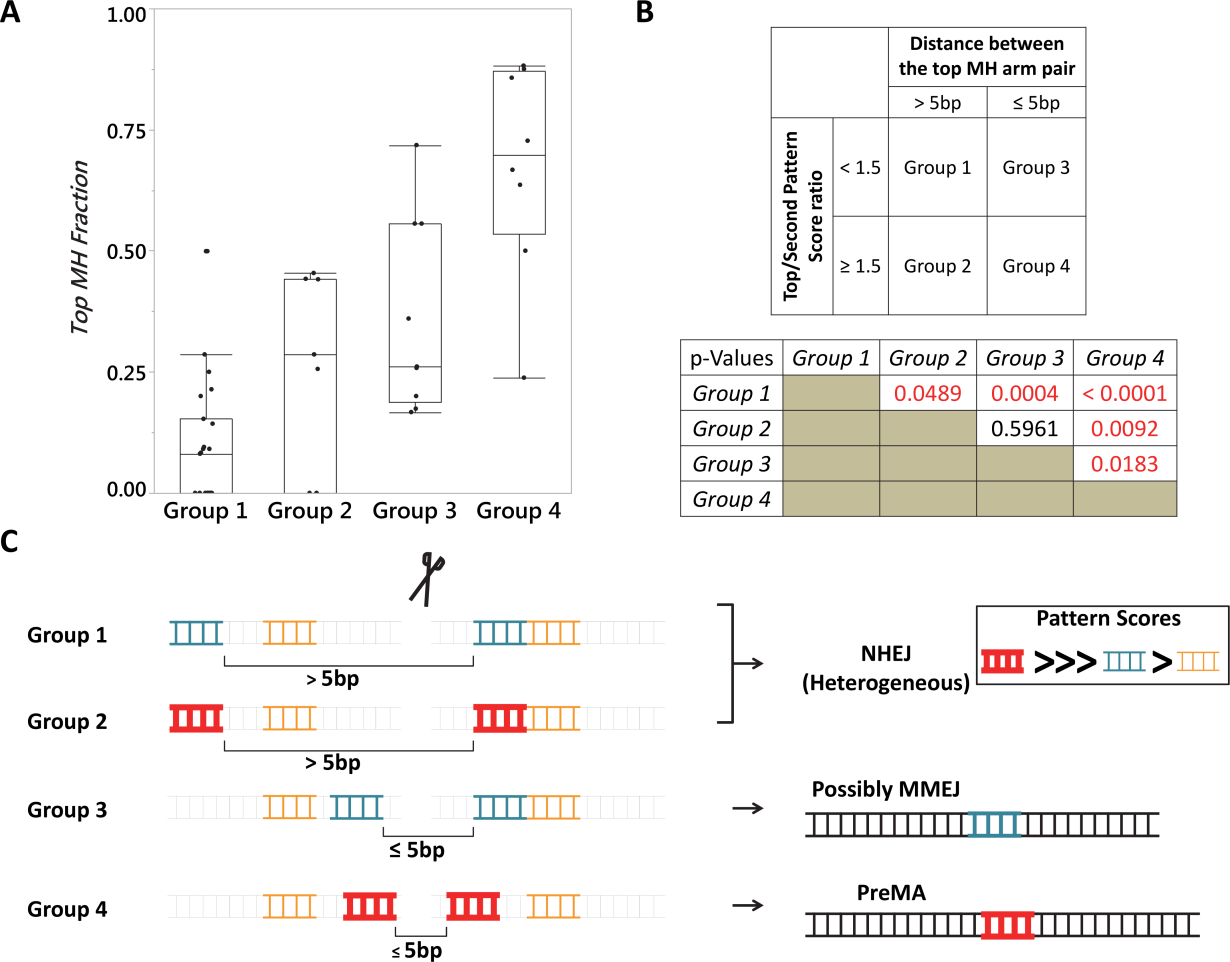
Competition hypothesis version 2. **A.** Outlier plot summarizing repair outcomes from 47 genomic targets using TALEN and CRISPR-Cas9. Close proximity of top predicted MH arms (Groups 3 and 4) appears to be the primary determinant for generating PreMA type outcomes as no target from Groups 1 and 2 had *Top MH Fraction* exceeding 0.5. When the top predicted allele had at least 50% higher *Pattern Score* than the second predicted allele (Groups 2 and 4), it was a strong indicator for inducing MMEJ-class repairs. **B.** *Top* Definition for each of the 4 groups used in Panel A. Each and every zebrafish genomic locus was segmented into these categories. *Pattern scores* were derived using RGEN online tool. *Bottom* P-values calculated by Wilcoxon’s Each Pair Calculation (adjusted for multiple comparisons). **C.** Graphical representation of each group detailed in Panel A. Groups 1 and 2 are prone to activate NHEJ-type outcomes, presumably because the yet-unidentified MMEJ factor fails to localize to suitable microhomology arm pairs, limited by how far apart these arms are. Group 4 is most suitable for strong MMEJ activation because it satisfies the proximity requirement AND the relative strength requirement. The latter may aid in the kinetics of the yet-unidentified MMEJ factor binding to the microhomology arms. Our data suggest that Group 3 is an intermediate group in terms of MMEJ activation. Perhaps extragenetic factors, such as cell cycle and epigenetic status may determine how favorable the loci are for MMEJ inductions.

These data implicate the utility of PreMA reagents for various applications that require precision gene editing. However, sgRNA design based on the Competition Hypothesis only yielded 5 PreMA reagents out of 20 that were tested (**S3 Table**, **S3 Note**). While this represented an improvement over the initial approach solely relying on the *Microhomology Score* (1 out of 14; **S1 Table**), we sought to further fine-tune the predictability for the PreMA targets. To this end, we pooled the results from all the programmable nucleases described above (**S1 and S3 Tables**) and seven *Medium* ~ *High Competition* sgRNAs designed as controls based on the Competition Hypothesis (**S4 Table**). In so doing, we noted that PreMA outcomes were only observed if the two arms of the top predicted microhomology were separated by no more than 5 bp. We subsequently identified the second parameter: high ratio (≥ 1.5) of the *Pattern Scores* between the top predicted and second predicted MMEJ alleles for a given locus (**Fig 7**). Seven out of eight reagents that satisfied both of these parameters were PreMA. Of the nine reagents that satisfied the first parameter but not the second, two were PreMA. All the other thirty reagents that failed to meet the first parameter failed to induce the top predicted MMEJ allele strongly. Most importantly, all the failed cases (i.e., incorrect predictions according to the original Competition Hypothesis) can be explained using our revised approach (Competition Hypothesis V2; **Fig 7C**). The Version 2 also captured three PreMA reagents that would have been missed by the original Competition Hypothesis alone, and one PreMA reagent that would have been missed by the *Microhomology Score* alone. Similar trends were observed using independently collected, previously published deep sequencing dataset from zebrafish [28] and HeLa cells [14] (**S4 Fig**).

### Mechanism of MMEJ-activation may be conserved in vertebrates

**Fig 8:**
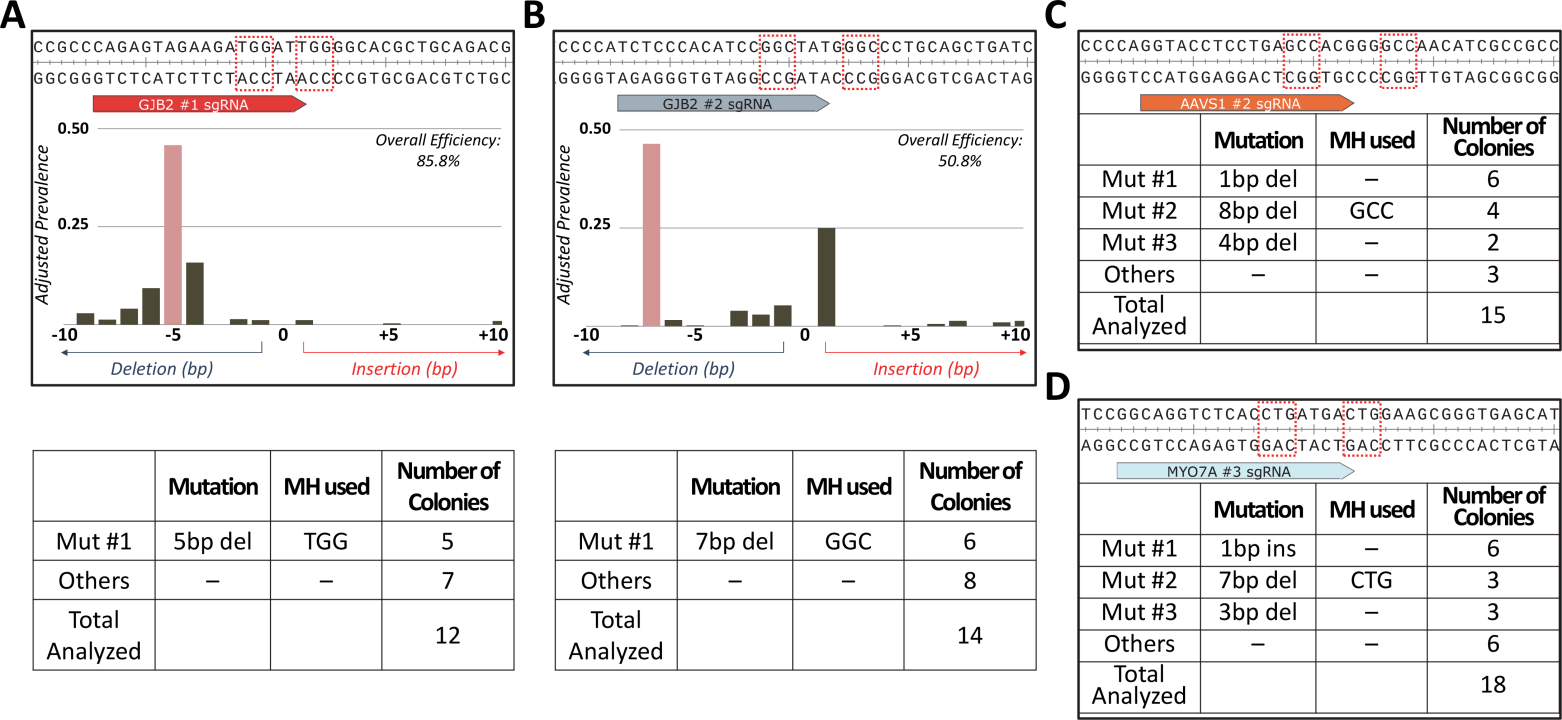
Competition hypothesis V2 targets trigger primary repair by MMEJ in HEK293T cells. **A** & **B.** *Top–*Wildtype human *GJB2* sequences with sgRNA target sites annotated. The dotted red boxes denote the top predicted MH arms. Summary TIDE analysis outcomes are also presented showing ~ 45% *Top MH Fractions* for *GJB2* #1 and #2 sgRNA. Red bar indicates the predicted deletion allele. Calculations for *Adjusted Prevalence* conform to calculations for *Top MH Fractions* detailed in **S3 Note**. *Bottom–*summary data from subcloning analyses for *GJB2* #1 sgRNA (**A**) and #2 sgRNA (**B**). **C** & **D.** *Top–*Wildtype human *AAVS1* and *MYO7A* sequences with sgRNA target sites annotated. The dotted red boxes denote the top predicted MH arms. *Bottom–*summary data from subcloning analyses for *AAVS1* #2 sgRNA (**A**) and *MYO7A* #3 sgRNA (**B**).

To test the generalizability of our findings, we prospectively designed 11 sgRNAs against the human genome (**S5 Table**) and delivered as RNPs to HEK293T cells. Of the 5 active guides cutting above 20% efficiency, DSBs induced by *GJB2* #1 and #2 guides resulted in more homogeneous repair outcomes (**Fig 8A** and 8**B**) than any of the 92 guides tested by Bae, *et al* (**S4B Fig**) [14]. DSBs at *AAVS1* #2 and *MYO7A* #3, on the other hand, repaired primarily through 1bp indels, consistent with the report by Bae, *et al* using HeLa cells. Intriguingly, the second most prevalent class of repair at these loci was the top predicted MMEJ allele (**Fig 8C** and 8**D**), as identified by subcloning analyses. We thus conclude that the specific trigger for efficient MMEJ-activation may be conserved in vertebrate organisms, albeit with nuances that are yet to be elucidated.

### Accessing the PreMA algorithm through MENTHU (MMEJ kNockout target heuristic utility)

The broad potential utility of this updated PreMA Algorithm for MMEJ prediction led us to develop a web-based automated analysis tool called MENTHU (http://genesculpt.org/menthu/). The tool can also be downloaded and installed on a local computer (www.github.com/Dobbs-Lab/menthu/). MENTHU accepts a user-specified DNA sequence and targeting scheme as input, and outputs recommended CRISPR gRNA target sites that are predicted to result in PreMA type outcomes. We validated the accuracy and functionality of MENTHU against select gRNA sites used in this study using whole exonic sequences as inputs (**S6 Table**); importantly, the software identified novel PreMA candidate loci against *surf1* and *tdgf1* where only Group 3 gRNA loci had been found by previous methods. Finally, we conducted a preliminary assessment to examine the prevalence of PreMA loci and found roughly 10% prevalence of such loci among all possible NGG PAM on human *CSF2* as well as zebrafish *tp53* genes (**S7 Table**).

## Discussion

To date, precision genome engineering is limited by the ability to predictably, efficiently, and reproducibly induce the identical sequence alterations in each and every cell. Here, we demonstrate the feasibility and utility of creating allelic consistency by an MMEJ-centric approach for designing programmable nucleases. While the precise cellular components of the molecular machinery involved in MMEJ remain incompletely understood [8], we provide evidence that we can enrich for MMEJ events by strictly sequence-based queries.

We also demonstrate that MMEJ predominant repairs do not operate at the cost of overall mutagenic efficiency; median edit efficiency for PreMA reagents was 91.4% in zebrafish. As genetically unaltered wildtype zebrafish were used throughout the study, we have no reason to believe that NHEJ should have failed at any tested loci. This is in contrast to the proposal that MMEJ is a back-up pathway to NHEJ [7, 8, 16, 17, 29]. Our findings, on the other hand, are compatible with a previous report wherein MMEJ-specific factors such as PolQ are abundantly expressed in embryonic zebrafish [20]. Interestingly, maternally zygotic PolQ mutant embryos failed to repair DSB at two out of three MMEJ loci, leading to premature deaths [20]. The third locus–which preferentially used a 2 bp microhomology and exhibited more heterogeneous DSB repair outcomes–was able to be repaired at a measurable rate, though significantly less so than in WT embryos. Thus NHEJ and MMEJ may be non-competing, parallel processes with unique triggers.

Based on the data presented here, we speculate that there is a reaction-limiting factor for MMEJ that is involved in identifying compatible microhomology pairs on both sides of the DNA double stranded break. In the case of abundantly available local microhomology pairs, this factor may fail to localize to a single suitable pair, thus rejecting the MMEJ activation. As end-resection is required for MMEJ and not for NHEJ [9, 17, 18], this yet identified factor may be the deciding factor for committing DSB repair through one End Joining pathway to another. This view is similar to a recent report wherein CtIP/Artemis dependent limited end resection was a key trigger for a slow-kinetic Lig1/3 independent NHEJ event that frequently utilized Microhomology to repair a reporter plasmid [30]. In our analysis, the primary driver of this decision making process is the proximity of 2 microhomology arms, further aided by the lack of competing microhomology arms.

Successful deployment of the PreMA reagents makes it possible to directly dictate the reading frame or to do in-frame gene manipulations on endogenous targets. Even assuming a somewhat modest outcome of 50% edit efficiency in which 50% of the mutant allele pool is of the desirable allele, more than 10% of the cell population will be homozygous for this desired allele. Conversely, many real-life gene editing applications would require only one of the diploid copies to be corrected. In these settings under the same assumptions, just 11 viable cells are needed to achieve 95% confidence for establishing the right clone, bringing the idea of precision molecular surgery closer to reality.

Our present study expands upon the current state-of-art understanding for MMEJ activation and demonstrates the ability to prospectively design robustly active PreMA reagents *in-vivo*. We also provide evidence that this 2-component approach may be broadly applicable beyond zebrafish; testing of the true generalizability of our approach will be facilitated by our web-based application, MENTHU (http://genesculpt.org/menthu/). Importantly, MENTHU allows users to flexibly define a PAM sequence and the cut site (in nts from PAM) so as to accommodate potential future variants of the CRISPR system. Active investigations are underway to accommodate alternative or more lax PAM requirements, such as the case with xCas9–a recently described variant of Cas9 that may function efficiently on an NG PAM [31]. As MMEJ-based loci are inherently restricted to genomic locations that leverage endogenous sequence contexts, availability of more flexible programmable nucleases will become the key for broadening the utility of PreMA reagents.

We provide strong evidence to support the utility of the MMEJ-centric approach beyond phenotype-genotype correlations in F0 animals. We envision this approach to be useful for: 1) studying the effects of homozygous gene knock-out in culture cells (as opposed to more common, compound heterozygous loss-of-function cell lines), 2) rapid small molecule screening in F0 animals as a complimentary approach to studying in germline mutant animals, 3) globally sharing and reproducing gene knock-out cell and animal lines, 4) pathway dissection for MMEJ, and finally, 5) human gene therapy.

## Materials and methods

### Ethics statement

The animal studies were conducted following guidelines and standard procedures established by the Mayo Clinic Institutional Animal Care and Use Committee (Mayo IACUC). The Mayo IACUC approved all protocols involving live vertebrate animals (A23107, A 21710 and A34513).

### Microhomology arms

For the purpose of this study, microhomology is defined as any endogenous direct sequence repeats of ≥ 3 bp surrounding a DSB site. ≤ 2 bp direct sequence repeats were not considered sufficient substrates of MMEJ activation based on our initial analyses of the DSB repair outcomes by previously designed programmable nucleases. Correlation for *Microhomology Fraction* vs the *Slope Value* was tangentially stronger when only ≥ 3 bp arms were considered (r^2^ = 0.382 vs r^2^ = 0.353; **S1 Fig**) in zebrafish, whereas the correlation was lost when 2 bp arms were considered in HeLa cells (r^2^ = 0.339 vs r^2^ = 0.034; **S2 Fig**).

### Zebrafish husbandry

All zebrafish (*Danio rerio*) were maintained in accordance with protocols approved by the Institutional Animal Care and Use Committee at Mayo Clinic. Zebrafish pairwise breeding was set up one day before microinjections and dividers were removed the following morning. Following microinjections, the fertilized eggs were transferred to Petri dishes with E3 media [5 mM NaCl, 0.17 mM KCl, 0.33 mM CaCl_2_, and 0.33 mM MgSO_4_ at pH 7.4] and incubated at 28.5 °C. All subsequent assays were conducted on fish less than 3 dpf, with the exception of assessing for germline transmission. In this case, injected founders were raised to adulthood per the standard zebrafish husbandry protocol.

### DNA oligonucleotide preparation

All of the oligonucleotides used for this study were purchased from IDT (San Jose, CA). Upon arrival, they were reconstituted into 100μM suspensions in 1x TE and stored at -20 °C until use.

### sgRNA expression vector synthesis

pT7-gRNA was a gift from Wenbiao Chen (Addgene plasmid # 46759). Given that the minimum requirement for the T7 promoter is a single 5’ G, the GG start on this vector was mutagenized via site-directed mutagenesis (SDM) to accommodate GA, GC, GT starts, using Forward and Reverse primers given (**S8 Table**). Platinum Pfx DNA Polymerase (Invitrogen 11708013. Carlsbad, CA) was used for 20 cycles of PCR amplification with the Tm of 60 °C and extension time of 3 minutes. DpnI (NEB R0176. Ipswich, MA) was subsequently added to reaction prior to transforming DH5α cells. The target sequence was cloned in as previously described, with the exception of conducting oligo annealing and T4 ligation (NEB M0202. Ipswich, MA) in 2 separate steps. In each case, transformed cells were cultured with Carbenicillin, and plasmids were purified with Plasmid Mini Kit (Qiagen 12123. Hilden, Germany).

### TALEN synthesis

TALEN constructs were generated using the FusX kit (Addgene # 1000000063) as previously described [32]. In short, RCIscript-GoldyTALEN was linearized with BsmBI (NEB R0580. Ipswich, MA) along with 6 triplet RVD (Repeat-Variable Diresidue) plasmids. Subsequently, they were ligated together in one reaction by a modified Golden-Gate Assembly. Blue-White colony screening with X-Gal/IPTG, colony PCR and finally pDNA sequencing were done to ascertain the correct assembly.

### In-vitro transcription and RNA preparation

pT3TS-nCas9n (a gift from Wenbiao Chen: Addgene plasmid # 46757) was linearized with XbaI (NEB R0145. Ipswich, MA), whereas TALEN constructs were linearized with SacI-HF (NEB R3156. Ipswich, MA) and sgRNA vector with BamHI-HF (NEB R3136. Ipswich, MA). Tyr sgRNA #2 –a construct made in the Essner Lab–was linearized with HindIII (NEB R0104. Ipswich, MA). RNA was made using T3 mMessage mMachine kit (Ambion AM1348. Foster City, CA) or HiScribe T7 High Yield RNA synthesis kit (NEB E2040. Ipswich, MA) according to manufacturer’s protocols with the addition of RNA Secure to the reaction (Ambion AM7010. Foster City, CA). To purify RNA, phenol-chloroform extraction was performed using Acid Phenol, Chloroform, and MaXtract High Density Tubes (Qiagen 129046. Hilden, Germany). RNA was then precipitated with Isopropanol at -20 °C, pelleted, air dried and resuspended into nuclease free water. The quality and quantity of RNA were ascertained by using a Nanodrop spectrophotometer and running aliquot on agarose gel. Each batch of RNA was aliquoted into small single use tubes and stored at -80 °C until the morning of microinjections.

### CRISPR-Cas9 RNP preparation for microinjections

sgRNA was thawed on ice in the morning of microinjections. This was then diluted to the concentration of 300 ng/μL in Duplex Buffer [100 mM KCH_3_COO, 30 mM HEPES at pH 7.5]. Appropriate folding of sgRNA was induced by heating it to 95 °C for 5 minutes and gradually cooling the solution to room temperature. Equal volumes of sgRNA and 0.66 mg/mL Alt-R S.p. Cas9 Nuclease 3NLS (IDT 1074181. San Jose, CA) in Cas9 Working Buffer [20 mM HEPES, 100 mM NaCl, 5 mM MgCl_2_, 0.1 mM EDTA at pH 6.5] were mixed and incubated at 37 °C for 10 minutes. RNP solutions were subsequently kept on ice until immediately before use.

### TALEN and CRISPR-Cas9 RNA preparation for microinjections

RNA was thawed on ice in the morning of microinjections. TALEN mRNA was diluted to working concentrations in the range of 12.5 ng/μL to 100 ng/μL in Danieau solution [58 mM NaCl, 0.7 mM KCl, 0.4 mM MgSO_4_, 0.6 mM Ca(NO_3_)_2_, 5.0 mM HEPES at pH 7.6]. sgRNA and nCas9n mRNA were mixed and diluted to the final concentrations of 150 ng/μL and 100 ng/μL, respectively, in Danieau solution. These were all kept on wet ice until immediately before use.

### Microinjections

Microinjections were carried out as previously described [33]. In short, 1-cell stage fertilized embryos were harvested and aligned on an agarose plate with E3 media. In the case of CRISPR-Cas9 reagents, either 1 or 2 nL was delivered to the cell. In the case of TALEN reagents, 1 ~ 3 nL was delivered to the yolk mass. They were then transferred to Petri dishes in E3 media for incubation at 28.5 °C. Dead and/or nonviable embryos were counted and removed each subsequent morning.

### Phenotype scoring

Each experiment was conducted in at least a technical triplicate and a biological duplicate. Detailed outcomes are provided in **S4 Table**. Gross phenotypes were scored visually on either 1 dpf or 2 dpf using a standard dissecting microscope. Subsequently, representative pictures were taken with Lightsheet Z.1 (Zeiss 2583000135. Oberkochen, Germany). Shortening Fractions were scored as previously reported [34]. In short, live 2 dpf larvae were immobilized and positioned in 3% methylcellulose. An Amscope camera (MU1403. Irvine, CA) mounted on a Leica Microscope (M165. Wetzlar, Germany) was used to capture a 15 second clip of the beating heart at 66 fps. These clips were subsequently used to measure the distance of the long axis along the ventricle at maximum dilation and maximum contraction using ImageJ software [35]. Shortening Fraction was calculated as below:

$$Shortening\;Fraction=100*(1-\frac{Distance\;at\;Maximum\;Shortening}{Distance\;at\;Maximum\;Dilation})$$

Shortening Fractions from 5 cycles were averaged for each animal.

### Zebrafish DNA extraction and assessing mutagenic outcomes

Typically, 8 uninjected wildtype fish and 8 injected fish were randomly collected without prior screening for phenotype. Chorion was predigested with 1 mg/mL Pronase at room temperature as needed. 1 ~ 3 dpf animals were then sacrificed for individual DNA extractions in 100 mM NaOH for 15 minutes at 95 °C. Equal volumes of 8 fish DNA from the same condition were then mixed and used as templates for PCR with either MyTaq (Bioline BIO-21108. London, UK), Phusion (NEB M0530. Ipswich, MA), or KOD (EMD Millipore 71085. Burlington, MA) polymerases per manufacturer’s protocols. The PCR amplicon was resolved on agarose gel, gel extracted with either Monarch DNA Gel Extraction Kit (NEB T1020. Ipswich, MA) or QiaEx II Gel Extraction Kit (Qiagen 20021. Hilden, Germany), and subsequently sent out for sequencing. The chromatograms from both uninjected and injected amplicons were used for TIDE analysis [23]. Alternatively, purified amplicons were used for subcloning analysis with either Topo-TA Cloning Kit (Thermo Fisher Scientific 451641. Waltham, MA) or StrataClone PCR Cloning Kit (Agilent 240205. Santa Clara, CA) per manufacturer’s protocols. Resultant white to pale blue colonies by Blue-White screening were subjected to colony PCR with M13F and R primers, using MyTaq polymerase. Once successful amplification was confirmed on agarose gel, these amplicons were sent out for sequencing either with M13F, M13R or endogenous gene target primers.

### Germline transmission for 5 bp deletion generated by N2B sgRNA #1

RNP containing N2B sgRNA #1 was prepared at 4x diluted dose as described above. Following microinjections, viable fish were raised to sexual maturity. Both F0 founders we attempted to out cross successfully mated and produced viable embryos. DNA was extracted from all viable embryos on 1 dpf, and individual DNA was used as template for PCR amplification using MyTaq Polymerase. Once the thermocycling ran to completion, the amplicons were melted by heating to 95 °C and re-annealed by a gradual step-wise cooling. Surveyor assay [27] was conducted per the manufacturer’s protocol (IDT 706025. San Jose, CA), and the results were analyzed by resolving the post-digest amplicons on agarose gel. Amplicons from 4 heterozygous offspring each were subcloned, and 5 colonies each were sent for Sanger Sequencing to confirm successful transmission of the 5 bp deletion allele.

### Reanalyses of previously published deep sequencing dataset

For zebrafish dataset, sgRNA screen SRA files were obtained from NCBI’s Short Read Archive (Accession: PRJNA245510) [28]. These files were converted to the fastq format with fastq-dump command using—*split-spot* function under SRA Toolkit (NCBI. Bethesda, MD). The fastq files were then uploaded onto Cas-Analyzer (http://www.rgenome.net/cas-analyzer/) and analyzed with Comparison range of 25 ~ 40 and Minimum frequency of 1 [36]. Following number of reads were recorded: total, total mutant, total top predicted allele. A top predicted allele was allowed to be included so long as the read contained no more than 2 polymorphisms on the analysis window AND the polymorphisms did not fall on the microhomology arms. Subsequently, the calculated mutagenic efficiency was plotted against the reported efficiency (r^2^ = 0.306). Of 122 targets designed by Gangnon, *et al*, following were excluded to arrive to the 34 targets that were used for analysis presented in **S4 Fig** Panel A: non-NGG targets (36 loci), targets that did not align to WT consensus sequence (GRCz11; 8 loci), targets with total recovered read counts less than 1% of expected (7 loci), high rate of permutation outside of the target site (1 locus), targets that did not have good agreements between calculated and reported (i.e., fell beyond 99% Confidence Interval; 10 loci), targets that had less than 5% calculated AND reported mutagenic efficiencies (26 loci).

The HeLa cell dataset [14] was obtained from Dr. Kim in the form of excel spread sheet with aligned sequence outputs +/- 25 bp of the predicted cut site. Following number of reads were recorded: total, total mutant, total top predicted allele with 2 bp microhomology, and total top predicted allele with 3 bp or longer microhomology. As with zebrafish dataset, top predicted allele was allowed to be included so long as the read contained no more than 2 polymorphisms on the analysis window AND the polymorphisms did not fall on the microhomology arms. Of the 92 targets, following were removed to arrive to 74 targets that were used for analyses presented in **S2 Fig** and **S4 Fig** Panel B: targets with total recovered read counts less than 1% of expected (2 loci), and targets that had less than 20% mutagenic efficiency (16 loci). There were no targets with non-NGG PAM, no alignment against consensus sequence, nor a high rate of permutation outside of the predicted cut site.

### Cell culture and RNP transfection

HEK293T cell line was purchased from ATCC (Manassas, VA) and maintained in DMEM (Invitrogen. Carlsbad, CA) with 10% Fetal Bovine Serum (Sigma. St. Louis, MO). DAPI stain was used to check for mycoplasma contamination.

RNP transfection was conducted as follows in a 48-well format using Lipofectamine CRISPRMAX reagent (Invitrogen CMAX00015. Carlsbad, CA). *In vitro* transcribed sgRNA was diluted to 2 μM concentration in Duplex Buffer. Secondary structure was induced by heating it to 95 °C for 5 minutes and gradually cooling it to room temperature. 3.0 μL of sgRNA was then complexed with 3.0 μL of 2 μM Alt-R S.p. Cas9 Nuclease V3 (IDT 1081058. San Jose, CA) in 42.8 μL OPTI-MEM (Life Technologies. Carlsbad,CA) and 1.2 μL Cas9 Plus Reagent. This mixture was incubated for 5 minutes at 25 °C. 2.4 μL of CRISPRMAX reagent and 47.6 μL OPTI-MEM was then added to the RNP, transferred to empty wells, and further incubated for 20 minutes at 25 °C. 200 μL cell suspension at 400,000 cells / mL in complete medium were subsequently added to each well. The dosing of RNP was consistent for all targets except for both *GJB2* targets wherein 1 μM each of sgRNA and Cas9 protein was used.

### HEK293T cell DNA extraction and assessing mutagenic outcomes

HEK293T cells were harvested 24 hour post transfection for gDNA extraction using DNeasy Blood & Tissue Kit (Qiagen 69506. Hilden, Germany). 20 ng of gDNA was used as a template for PCR with KOD polymerase per manufacturer’s protocol. The PCR amplicon was resolved on agarose gel, gel extracted with Monarch DNA Gel Extraction Kit and subsequently sent out for sequencing. The chromatograms from both uninjected and injected amplicons were used for TIDE analysis [23].

For sgRNAs that showed > 20% activity by TIDE, single A overhang was added to the 3’ end of purified amplicons by incubating them with MyTaq polymerase at 72 °C for 15 minutes. They were then used for subcloning analysis with StrataClone PCR Cloning Kit. 96 resultant white to pale blue colonies by Blue-White screening were subjected to colony PCR with endogenous primers using MyTaq polymerase. Once successful amplification was confirmed on agarose gel, these amplicons were subjected to T7E1 assay [27]. Briefly, 2.5 μL each of colony PCR amplicon and wildtype amplicon were heteroduplexed in 1x NEB 2.0 Buffer (25 μL). This was incubated for 15 minutes at 37 °C with 0.5 μL T7 Endonuclease I (NEB m3020. Ipswich, MA) and 4.5 μL dH2O. The digested amplicon was resolved on 2% agarose gel. Number of colony PCR-positive clones and digest positive clones are reported in **S5 Table**. Some of the digest positive clones were then sent for sequencing to ascertain the nature of mutation.

2 targets (*CSF2* #1 and *MYO7A* #4) that did not meet the 20% edit efficiency cutoff nonetheless produced statistically significant aberrant sequence peaks by TIDE analysis (p < 0.001). Summary outcomes for *Top MH Fraction* calculation based on estimated alleleic prevalence is given in **S5 Table**.

### MENTHU

We developed a software tool, MENTHU (MMEJ kNockout Target Heuristic Utility), to automate calculations required to implement the 2-component PreMA strategy: 1) identification of top predicted microhomology arms separated by ≤ 5 bp of intervening sequence, and 2) identification of “low competition” target sites (i.e., with a #1-ranked to #2-ranked Pattern Score ratio ≥ 1.5). We designed MENTHU to first compute two of same sequence-based parameters (*Pattern Score* and *Microhomology Score*) used in the algorithm of Bae, *et al*., (which are computed online by the RGEN online tool, http://www.rgenome.net) To do so, we used R [37] to re-implement and modify the original Python source code provided in S3 Fig of the original publication [14]. The MENTHU webserver operates under R version 3.4.1 and RShiny [38] v1.0.5. The MENTHU code was built through RStudio [39] v1.1.442. Details regarding specific R package versions, complete documentation and a full downloadable version of MENTHU for local installation are provided at www.github.com/Dobbs-Lab/menthu/. MENTHU v2.0 can be freely accessed online at http://genesculpt.org/menthu/.

To preliminarily assess the abundance of PreMA loci, MENTHU was locally run to screen the sequences of two genes: human colony stimulating factor 2 (*CSF2*; Gene ID– 1437) and zebrafish tumor protein p53 (*tp53*; Gene ID– 30590). MENTHU was run twice on each gene: exonic target screen and whole gene target screen. A custom R script was used to mine the MENTHU results in a .csv format to determine both the amounts of total targetable sites by spCas9 (i.e., total number of unique cut sites with NGG PAM on either strand) and the subset of those predicted to be PreMA.

### Statistical analyses

All of the statistical analyses were carried out using JMP software (SAS Institute. Cary, NC). In all instances, p-values were calculated assuming non-Gaussian Distributions. Wilcoxon Each Pair calculation was used for multiple group comparisons with adjusted p-values.

## Supporting information

S1 Fig Overabundance of Microhomology arms is a negative predictor of MMEJ activation in zebrafish. **A** Box plot showing the distribution of *Slope Values* across 19 zebrafish genomic targets. **B** Scatter plot of *MH Fraction* against *Slope Value*, focused only on microhomology arms of ≥ 3 bp. Linear fit with 95% Confidence Interval (shade) is shown. r^2^ = 0.382, p = 0.0048. **C** Scatter plot of *MH Fraction* against *Slope Value* including 2 bp microhomology arms. Linear fit with 95% Confidence Interval (shade) is shown. r^2^ = 0.353, p = 0.0073. *Pattern Scores* and *Microhomology Scores* were derived using RGEN online tool (http://www.rgenome.net).
(TIF)

S2 Fig Overabundance of Microhomology arms is a negative predictor of MMEJ activation in HeLa cell. **A** Scatter plot of *MH Fraction* against *Slope Value*, focused only on microhomology arms of ≥ 3 bp using the first 50, alphabetically sorted HeLa cell targets. Linear fit with 95% Confidence Interval (shade) is shown. r^2^ = 0.339, p = 0.0001. **B** Scatter plot of *MH Fraction* against *Slope Value* including microhomology arms of 2 bp using the first 50, alphabetically sorted HeLa cell targets. Linear fit with 95% Confidence Interval (shade) is shown. r^2^ = 0.034, p = 0.2644. **C** Box plot showing the distribution of *Slope Values* across the first 50, alphabetically sorted HeLa cell targets. **D** Box plot showing the *MH Fractions* for High and Low competition sites amongst the remaining 40 HeLa cell targets, focused only on microhomology arms of ≥ 3 bp. p = 0.011. Targets with < 20% overall edit efficiency were excluded in all panels. *Pattern Scores* and *Microhomology Scores* were derived using RGEN online tool (http://www.rgenome.net).
(TIF)

S3 Fig Microhomology allele generated by *ttn.2* N2B sgRNA #1 is germline transmitted. Agarose gel showing PCR amplicon post Surveyor digest. 752 bp band is the whole amplicon. The expected cleavage products due to mutations at the CRISPR site are denoted by yellow arrowheads. The red asterisk denotes positive digest band due to a background T -> A SNP at position 389 from the 5’ end of the amplicon. Heterozygous animals are bolded and underlined. Genotypes of the first 4 heterozygous progenies from each founder were ascertained by subcloning analyses.
(TIF)

S4 Fig Fitting Competition Hypothesis Version 2 using independently collected zebrafish dataset and HeLa cell dataset. **A** Outlier plot summarizing independently collected repair outcomes from 34 zebrafish targets. All three Group 4 targets as well as some Group 3 targets yielded PreMA outcomes, validating our own training dataset. Importantly, none of Groups 1 and 2 targets were of this class. **B** Outlier plot summarizing repair outcomes from 90 genomic targets using CRISPR-Cas9. Similar to the findings in zebrafish, close proximity of the top predicted MH arms (Groups 3 and 4) appears to be the primary determinant for utilizing this MH pair efficiently. When the top predicted allele had at least 50% higher *Pattern Score* than the second predicted allele (Groups 2 and 4), median *Top MH Fractions* trended higher compared to Group 1 and 3, respectively. P-values calculated by Wilcoxon’s Each Pair Calculation (adjusted for multiple comparisons). Targets with < 20% overall edit efficiency were excluded from analysis. *Pattern Scores* were derived using RGEN online tool (http://www.rgenome.net).
(TIF)

S1 Table List and summary mutagenic outcomes of TALEN and CRISPR-Cas9 reagents that were designed primarily using the Bae, *et al* algorithm [14]. Underlined & italicized bases in sgRNA sequence denote mismatched bases due to the promoter requirement. *Pattern Scores* and *Microhomology Scores* were derived using RGEN online tool (http://www.rgenome.net). MH: Microhomology, SC: Subcloning. * Reagents prospectively designed according to Bae, *et al* algorithm [14]. ^†^ No raw sequencing data were available. However, the outcome had been compiled into a table prior to conception of this study. ^‡^ Injected with sgRNA and Cas9 mRNA (150 pg and 100 pg, respectively). ^^^ Gift from Wenbiao Chen (addene # 46761).
(TIF)

S2 Table Summary gross phenotyping outcomes from PreMA reagent injections. For *tdgf1*, Experiments 1a and 1b correspond to technical replicates using WT 1 as reference, uninjected control. *chrd* and *tdgf1* phenotypes were scored on 1 dpf, whereas *tyr*, *ttn*.*2* N2B, *ttn*.*2* phenotypes were scored on 2 dpf.
(TIF)

S3 Table List and summary sequence outcomes of Low Competition sgRNA that were designed around the Competition Hypothesis. Underlined & Italicized bases in gRNA sequence denote mismatched bases due to the promoter requirement. *Pattern Scores* and *Microhomology Scores* were derived using RGEN online tool. MH: Microhomology, SC: Subcloning, TIDE: Tracking Indels by DEcomposition. ^†^ Injected RNP at the dose of 115 pg sgRNA and 245 pg Cas9 due to poor viability at higher doses.
(TIF)

S4 Table List and summary sequence outcomes of Medium ~ High Competition sgRNA that were designed around the Competition Hypothesis. Underlined & Italicized bases in sgRNA sequence denote mismatched bases due to the promoter requirement. *Pattern Scores* and *Microhomology Scores* were derived using RGEN online tool (http://www.rgenome.net). MH: Microhomology, SC: Subcloning, TIDE: Tracking Indels by Decomposition. ^†^ Injected RNP at the dose of 115 pg sgRNA and 245 pg Cas9 due to poor viability at higher doses.
(TIF)

S5 Table List and summary sequence outcomes of human CRISPR-Cas9 targets that were designed around the Competition Hypothesis V2. Underlined & Italicized bases in sgRNA sequence denote mismatched bases due to the T7 promoter requirement. For loci wherein mutagenic efficiency and/or *Top MH Fraction* was calculated based on subcloning results, number of mutant/top predicted allele colonies are given in numerator and the total number of colonies analyzed are given in the denominator. *Pattern Scores* and *Microhomology Scores* were derived using RGEN online tool (http://www.rgenome.net).
(TIF)

S6 Table Example MENTHU output from select CRISPR-Cas9 targets used in this study, focusing only on out-of-frame mutations. The output was obtained by using the entire target exon sequence with 40 bp intronic sequence each on both 5’ and 3’ ends. The MENTHU output provides a 3’ NGG PAM sequence for each gRNA targets (italicized and underlined). MENTHU gRNA outputs that matched the target sequences used in this study are bolded. *Criteria 1* and *2* refer to 1) if top predicted microhomology arm is separated by 5 bp or less, and 2) if the ratio of top to second predicted *Pattern Scores* is at least 1.5. MENTHU is programmed to terminate calculations if the target site is negative for *Criterion 1*. As a result, no gRNA sequence output is obtained for *chrd* #1 and *mitfa* #2. Importantly, in two instances (*surf1* and *tgdf1*) where we only had Group 3 reagents, novel candidate PreMA sites were identified. ^*^ Result obtained by adjusting the value for *Criterion 2* to 1.0 as these were Group 3 guides that, by definition, does not satisfy *Criterion 2* of 1.5 or higher. ^^^ in-frame mutation by the experimental design. ^†^ 16 other candidate loci identified on this 3771 bp exon; only a partial list for alternate loci is given. ^‡^ 16 other candidate loci identified on this 822 bp exon; only a partial list for alternate loci is given.
(TIF)

S7 Table Preliminary analyses on the prevalence of PreMA loci reveal that about 10% of the CRISPR-Cas9 targetable loci on both human *CSF2* and zebrafish *tp53* genes fall in this category. This holds true for both at the gene and exonic levels. As expected, roughly two thirds of the PreMA reagents are predicted to induce preferentially out-of-frame mutations.
(TIF)

S8 Table List of primers used in this study. All the primer sequences are provided in 5’ -> 3’ order. For *urod* Reverse primer, M13F primer sequence was added at the 5’ end of the endogenous target sequence (bolded and italicized). For SDM primers, intended point mutation is indicated by bold and italic. * No endogenous primer was used to sequence the genomic loci of interest.
(TIF)

S1 Note Calculation of *Microhomology Fraction*. (DOCX)

S2 Note Calculation of *Slope Values*. (DOCX)

S3 Note Calculation of *Top Microhomology Fraction*. (DOCX)

S1 Data Sanger sequencing file used for the study. Whole amplicon sequencing outcomes are deposited as .ab1 files for *chrd* TALEN and *tyr* #2, *tdgf1*, *ttn*.*2* N2B #1, *ttn*.*2* #2 sgRNA targets. Other sequencing outcomes, including those used for subcloning analyses are provided in the .fastq formats.
(ZIP)

## References

[1] Campbell JM, Hartjes KA, Nelson TJ, Xu X, Ekker SC. New and TALENted genome engineering toolbox. Circ Res. 2013;113(5):571–87. 10.1161/CIRCRESAHA.113.301765 ; PubMed Central PMCID: PMCPMC3965580. 23948583 PMC3965580

[2] Doudna JA, Charpentier E. Genome editing. The new frontier of genome engineering with CRISPR-Cas9. Science. 2014;346(6213):1258096 10.1126/science.1258096 . 25430774

[3] Lieber MR. The mechanism of double-strand DNA break repair by the nonhomologous DNA end-joining pathway. Annu Rev Biochem. 2010;79:181–211. 10.1146/annurev.biochem.052308.093131 ; PubMed Central PMCID: PMCPMC3079308. 20192759 PMC3079308

[4] Carroll D. Genome engineering with targetable nucleases. Annu Rev Biochem. 2014;83:409–39. 10.1146/annurev-biochem-060713-035418 . 24606144

[5] Jao LE, Wente SR, Chen W. Efficient multiplex biallelic zebrafish genome editing using a CRISPR nuclease system. Proc Natl Acad Sci U S A. 2013;110(34):13904–9. Epub 2013/08/07. 10.1073/pnas.1308335110 ; PubMed Central PMCID: PMC3752207. 23918387 PMC3752207

[6] Ata H, Clark KJ, Ekker SC. The zebrafish genome editing toolkit. Methods Cell Biol. 2016;135:149–70. 10.1016/bs.mcb.2016.04.023 . 27443924

[7] Boulton SJ, Jackson SP. Identification of a Saccharomyces cerevisiae Ku80 homologue: roles in DNA double strand break rejoining and in telomeric maintenance. Nucleic Acids Res. 1996;24(23):4639–48. Epub 1996/12/01. ; PubMed Central PMCID: PMC146307. 8972848 10.1093/nar/24.23.4639 PMC146307

[8] Seol JH, Shim EY, Lee SE. Microhomology-mediated end joining: Good, bad and ugly. Mutat Res. 2017 10.1016/j.mrfmmm.2017.07.002 . 28754468 PMC6477918

[9] Sharma S, Javadekar SM, Pandey M, Srivastava M, Kumari R, Raghavan SC. Homology and enzymatic requirements of microhomology-dependent alternative end joining. Cell Death Dis. 2015;6:e1697 Epub 2015/03/20. 10.1038/cddis.2015.58 ; PubMed Central PMCID: PMC4385936. 25789972 PMC4385936

[10] Kent T, Chandramouly G, McDevitt SM, Ozdemir AY, Pomerantz RT. Mechanism of microhomology-mediated end-joining promoted by human DNA polymerase theta. Nat Struct Mol Biol. 2015;22(3):230–7. Epub 2015/02/03. 10.1038/nsmb.2961 ; PubMed Central PMCID: PMC4351179. 25643323 PMC4351179

[11] Nakade S, Tsubota T, Sakane Y, Kume S, Sakamoto N, Obara M, et al Microhomology-mediated end-joining-dependent integration of donor DNA in cells and animals using TALENs and CRISPR/Cas9. Nat Commun. 2014;5:5560 10.1038/ncomms6560 ; PubMed Central PMCID: PMCPMC4263139. 25410609 PMC4263139

[12] Yao X, Wang X, Liu J, Hu X, Shi L, Shen X, et al CRISPR/Cas9—Mediated Precise Targeted Integration In Vivo Using a Double Cut Donor with Short Homology Arms. EBioMedicine. 2017;20:19–26. 10.1016/j.ebiom.2017.05.015 ; PubMed Central PMCID: PMCPMC5478232. 28527830 PMC5478232

[13] Hisano Y, Sakuma T, Nakade S, Ohga R, Ota S, Okamoto H, et al Precise in-frame integration of exogenous DNA mediated by CRISPR/Cas9 system in zebrafish. Sci Rep. 2015;5:8841 10.1038/srep08841 ; PubMed Central PMCID: PMCPMC4350073. 25740433 PMC4350073

[14] Bae S, Kweon J, Kim HS, Kim JS. Microhomology-based choice of Cas9 nuclease target sites. Nat Methods. 2014;11(7):705–6. Epub 2014/06/28. 10.1038/nmeth.3015 . 24972169

[15] Qi Z, Redding S, Lee JY, Gibb B, Kwon Y, Niu H, et al DNA sequence alignment by microhomology sampling during homologous recombination. Cell. 2015;160(5):856–69. Epub 2015/02/17. 10.1016/j.cell.2015.01.029 ; PubMed Central PMCID: PMC4344887. 25684365 PMC4344887

[16] Corneo B, Wendland RL, Deriano L, Cui X, Klein IA, Wong SY, et al Rag mutations reveal robust alternative end joining. Nature. 2007;449(7161):483–6. Epub 2007/09/28. 10.1038/nature06168 . 17898768

[17] Truong LN, Li Y, Shi LZ, Hwang PY, He J, Wang H, et al Microhomology-mediated End Joining and Homologous Recombination share the initial end resection step to repair DNA double-strand breaks in mammalian cells. Proc Natl Acad Sci U S A. 2013;110(19):7720–5. Epub 2013/04/24. 10.1073/pnas.1213431110 ; PubMed Central PMCID: PMC3651503. 23610439 PMC3651503

[18] Zha S, Boboila C, Alt FW. Mre11: roles in DNA repair beyond homologous recombination. Nat Struct Mol Biol. 2009;16(8):798–800. Epub 2009/08/06. 10.1038/nsmb0809-798 . 19654615

[19] He MD, Zhang FH, Wang HL, Wang HP, Zhu ZY, Sun YH. Efficient ligase 3-dependent microhomology-mediated end joining repair of DNA double-strand breaks in zebrafish embryos. Mutat Res. 2015;780:86–96. Epub 2015/09/01. 10.1016/j.mrfmmm.2015.08.004 . 26318124

[20] Thyme SB, Schier AF. Polq-Mediated End Joining Is Essential for Surviving DNA Double-Strand Breaks during Early Zebrafish Development. Cell Rep. 2016;15(7):1611–3. 10.1016/j.celrep.2016.04.089 . 27192698

[21] Schulte-Merker S, Lee KJ, McMahon AP, Hammerschmidt M. The zebrafish organizer requires chordino. Nature. 1997;387(6636):862–3. 10.1038/43092 . 9202118

[22] Page-McCaw PS, Chung SC, Muto A, Roeser T, Staub W, Finger-Baier KC, et al Retinal network adaptation to bright light requires tyrosinase. Nat Neurosci. 2004;7(12):1329–36. 10.1038/nn1344 . 15516923

[23] Brinkman EK, Chen T, Amendola M, van Steensel B. Easy quantitative assessment of genome editing by sequence trace decomposition. Nucleic Acids Res. 2014;42(22):e168 10.1093/nar/gku936 ; PubMed Central PMCID: PMCPMC4267669. 25300484 PMC4267669

[24] Zhang J, Talbot WS, Schier AF. Positional cloning identifies zebrafish one-eyed pinhead as a permissive EGF-related ligand required during gastrulation. Cell. 1998;92(2):241–51. . 9458048 10.1016/s0092-8674(00)80918-6

[25] Seeley M, Huang W, Chen Z, Wolff WO, Lin X, Xu X. Depletion of zebrafish titin reduces cardiac contractility by disrupting the assembly of Z-discs and A-bands. Circ Res. 2007;100(2):238–45. Epub 2006/12/16. 10.1161/01.RES.0000255758.69821.b5 ; PubMed Central PMCID: PMC2756513. 17170364 PMC2756513

[26] Xu X, Meiler SE, Zhong TP, Mohideen M, Crossley DA, Burggren WW, et al Cardiomyopathy in zebrafish due to mutation in an alternatively spliced exon of titin. Nat Genet. 2002;30(2):205–9. Epub 2002/01/15. 10.1038/ng816 . 11788825

[27] Vouillot L, Thelie A, Pollet N. Comparison of T7E1 and surveyor mismatch cleavage assays to detect mutations triggered by engineered nucleases. G3 (Bethesda). 2015;5(3):407–15. 10.1534/g3.114.015834 ; PubMed Central PMCID: PMCPMC4349094. 25566793 PMC4349094

[28] Gagnon JA, Valen E, Thyme SB, Huang P, Akhmetova L, Pauli A, et al Efficient mutagenesis by Cas9 protein-mediated oligonucleotide insertion and large-scale assessment of single-guide RNAs. PLoS One. 2014;9(5):e98186 10.1371/journal.pone.0098186 ; PubMed Central PMCID: PMCPMC4038517. 24873830 PMC4038517

[29] Decottignies A. Microhomology-mediated end joining in fission yeast is repressed by pku70 and relies on genes involved in homologous recombination. Genetics. 2007;176(3):1403–15. Epub 2007/05/08. 10.1534/genetics.107.071621 ; PubMed Central PMCID: PMC1931558. 17483423 PMC1931558

[30] Biehs R, Steinlage M, Barton O, Juhasz S, Kunzel J, Spies J, et al DNA Double-Strand Break Resection Occurs during Non-homologous End Joining in G1 but Is Distinct from Resection during Homologous Recombination. Mol Cell. 2017;65(4):671–84 e5. 10.1016/j.molcel.2016.12.016 ; PubMed Central PMCID: PMCPMC5316416. 28132842 PMC5316416

[31] Hu JH, Miller SM, Geurts MH, Tang W, Chen L, Sun N, et al Evolved Cas9 variants with broad PAM compatibility and high DNA specificity. Nature. 2018 10.1038/nature26155 . 29512652 PMC5951633

[32] Ma AC, McNulty MS, Poshusta TL, Campbell JM, Martinez-Galvez G, Argue DP, et al FusX: A Rapid One-Step Transcription Activator-Like Effector Assembly System for Genome Science. Hum Gene Ther. 2016;27(6):451–63. 10.1089/hum.2015.172 ; PubMed Central PMCID: PMCPMC4931509. 26854857 PMC4931509

[33] Bill BR, Petzold AM, Clark KJ, Schimmenti LA, Ekker SC. A primer for morpholino use in zebrafish. Zebrafish. 2009;6(1):69–77. 10.1089/zeb.2008.0555 ; PubMed Central PMCID: PMCPMC2776066. 19374550 PMC2776066

[34] Hoage T, Ding Y, Xu X. Quantifying cardiac functions in embryonic and adult zebrafish. Methods Mol Biol. 2012;843:11–20. 10.1007/978-1-61779-523-7_2 ; PubMed Central PMCID: PMCPMC3762588. 22222517 PMC3762588

[35] Schneider CA, Rasband WS, Eliceiri KW. NIH Image to ImageJ: 25 years of image analysis. Nat Methods. 2012;9(7):671–5. ; PubMed Central PMCID: PMCPMC5554542. 22930834 10.1038/nmeth.2089 PMC5554542

[36] Park J, Lim K, Kim JS, Bae S. Cas-analyzer: an online tool for assessing genome editing results using NGS data. Bioinformatics. 2017;33(2):286–8. 10.1093/bioinformatics/btw561 ; PubMed Central PMCID: PMCPMC5254075. 27559154 PMC5254075

[37] Team. RC. R: A language and environment for statistical computing: R Foundation for Statistical Computing; 2016.

[38] Chang W CJ, Allaire JJ, Xie Y, and McPherson J. shiny: Web Application Framework for R. R package version 105. 2017.

[39] Team. R. RStudio: Integrated Development for R. 2016.

