# Supplementary figures and images for: Robust activation of microhomology-mediated end joining for precision gene editing applications
Source: PLoS Genet. 2018 Sep 12;14(9):e1007652. doi: 10.1371/journal.pgen.1007652 (PMC6152997; doi:10.1371/journal.pgen.1007652)

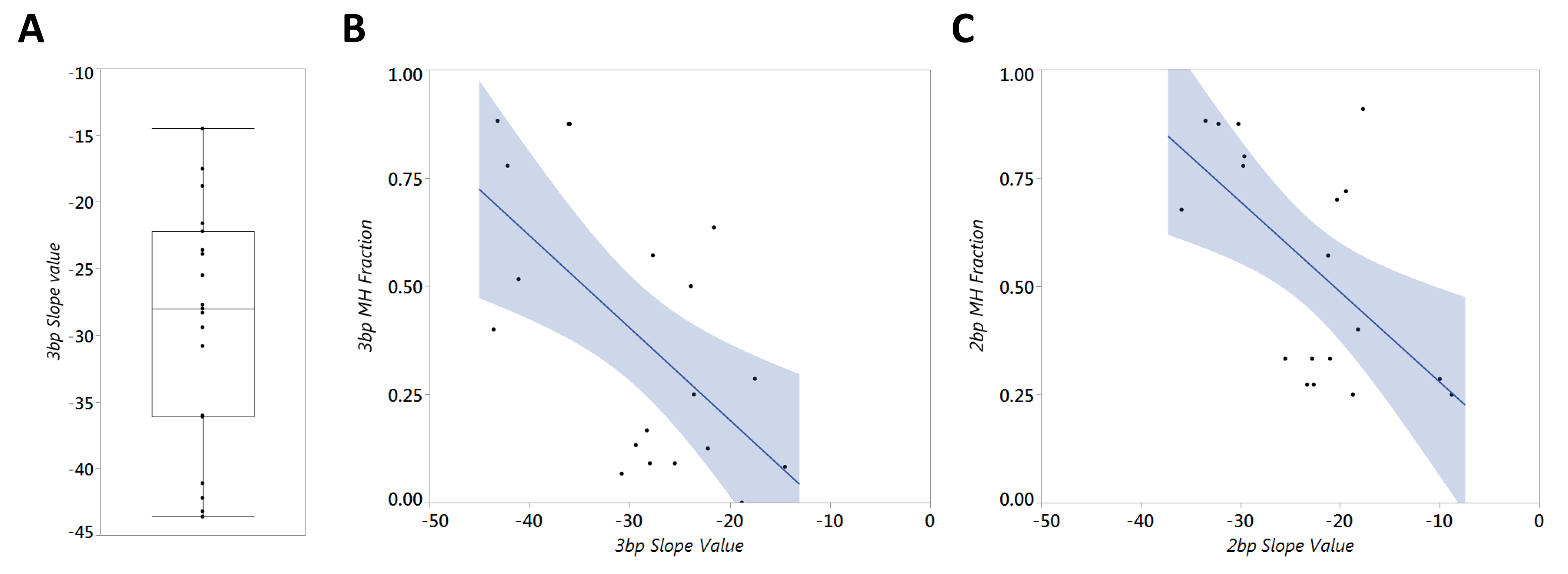

Supplement: S1 Fig — A Box plot showing the distribution of Slope Values across 19 zebrafish genomic targets. B Scatter plot of MH Fraction against Slope Value, focused only on microhomology arms of ≥ 3 bp. Linear fit with 95% Confidence Interval (shade) is shown. r2 = 0.382, p = 0.0048. C Scatter plot of MH Fraction against Slope Value including 2 bp microhomology arms. Linear fit with 95% Confidence Interval (shade) is shown. r2 = 0.353, p = 0.0073. Pattern Scores and Microhomology Scores were derived using RGEN online tool (http://www.rgenome.net). (TIF) [file pgen.1007652.s001.tif]

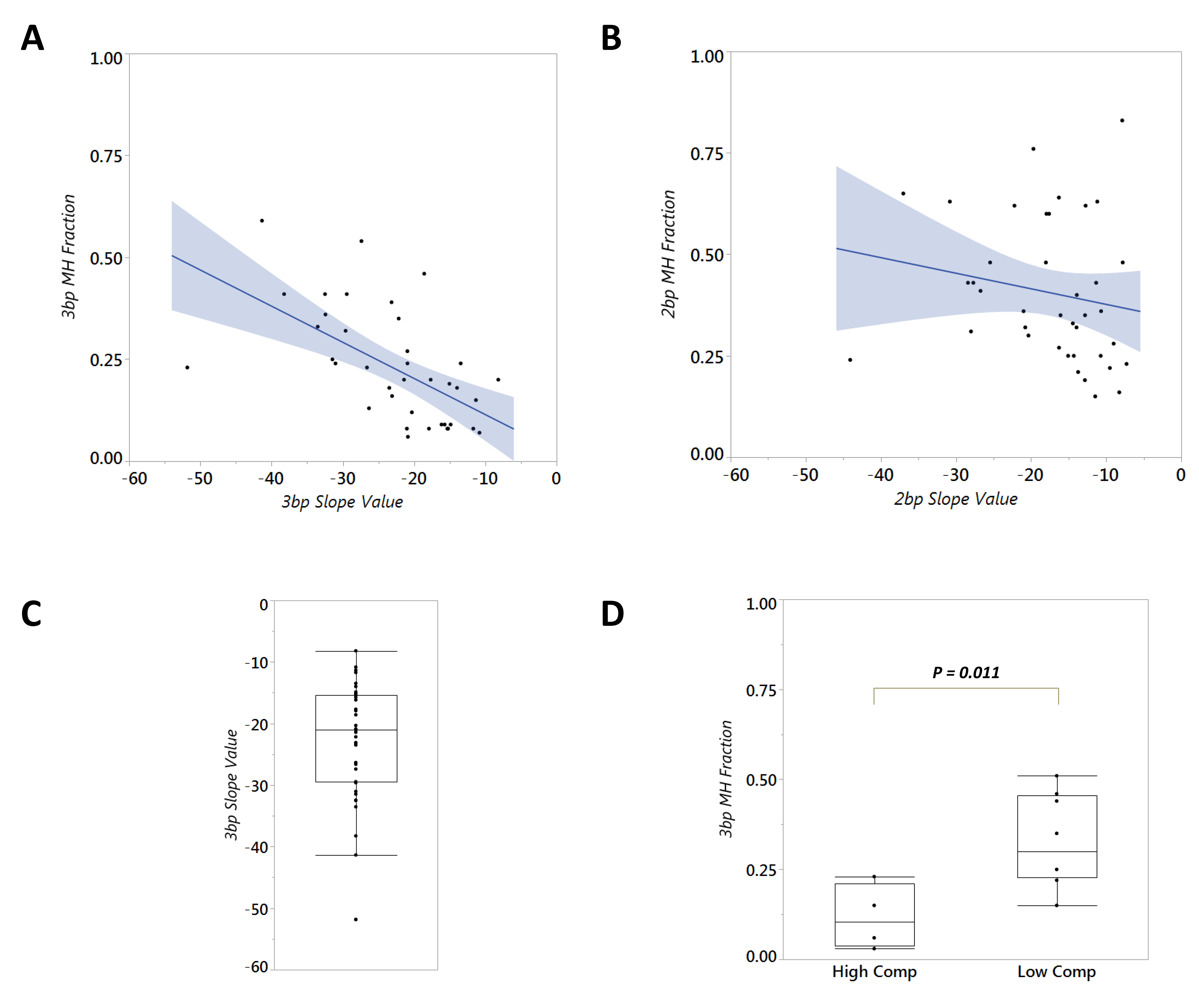

Supplement: S2 Fig — A Scatter plot of MH Fraction against Slope Value, focused only on microhomology arms of ≥ 3 bp using the first 50, alphabetically sorted HeLa cell targets. Linear fit with 95% Confidence Interval (shade) is shown. r2 = 0.339, p = 0.0001. B Scatter plot of MH Fraction against Slope Value including microhomology arms of 2 bp using the first 50, alphabetically sorted HeLa cell targets. Linear fit with 95% Confidence Interval (shade) is shown. r2 = 0.034, p = 0.2644. C Box plot showing the distribution of Slope Values across the first 50, alphabetically sorted HeLa cell targets. D Box plot showing the MH Fractions for High and Low competition sites amongst the remaining 40 HeLa cell targets, focused only on microhomology arms of ≥ 3 bp. p = 0.011. Targets with < 20% overall edit efficiency were excluded in all panels. Pattern Scores and Microhomology Scores were derived using RGEN online tool (http://www.rgenome.net). (TIF) [file pgen.1007652.s002.tif]

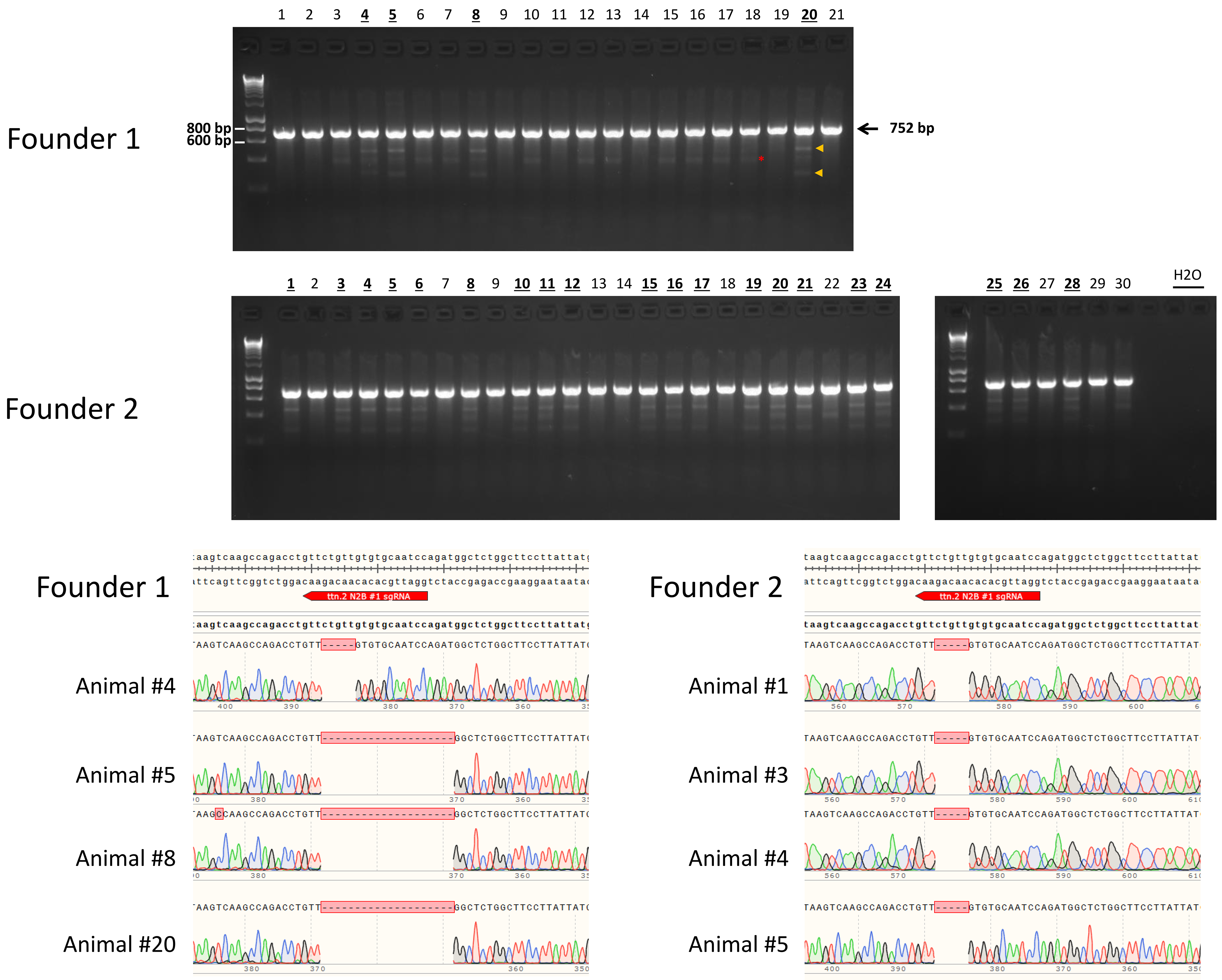

Supplement: S3 Fig — Agarose gel showing PCR amplicon post Surveyor digest. 752 bp band is the whole amplicon. The expected cleavage products due to mutations at the CRISPR site are denoted by yellow arrowheads. The red asterisk denotes positive digest band due to a background T -> A SNP at position 389 from the 5’ end of the amplicon. Heterozygous animals are bolded and underlined. Genotypes of the first 4 heterozygous progenies from each founder were ascertained by subcloning analyses. (TIF) [file pgen.1007652.s003.tif]

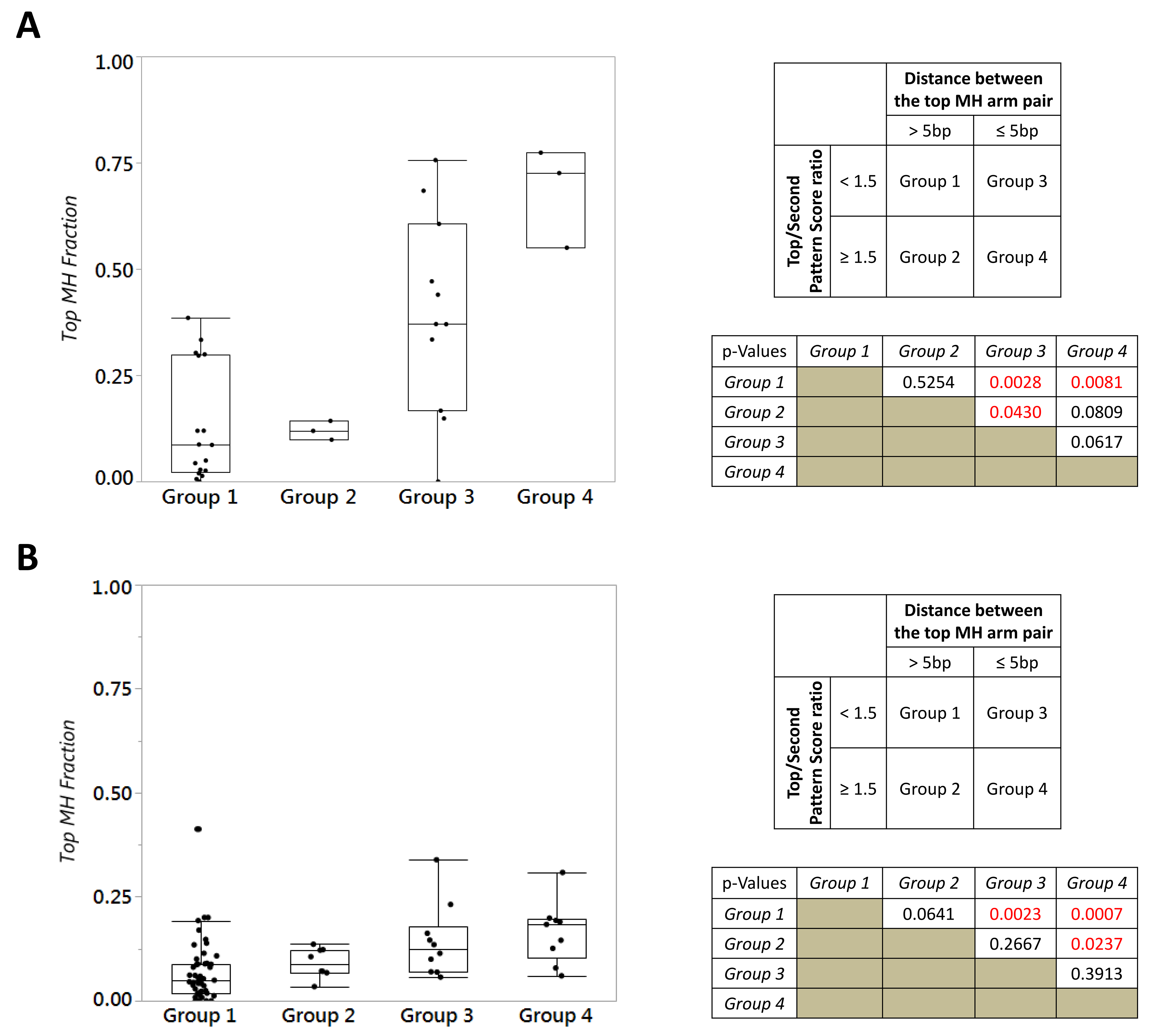

Supplement: S4 Fig — A Outlier plot summarizing independently collected repair outcomes from 34 zebrafish targets. All three Group 4 targets as well as some Group 3 targets yielded PreMA outcomes, validating our own training dataset. Importantly, none of Groups 1 and 2 targets were of this class. B Outlier plot summarizing repair outcomes from 90 genomic targets using CRISPR-Cas9. Similar to the findings in zebrafish, close proximity of the top predicted MH arms (Groups 3 and 4) appears to be the primary determinant for utilizing this MH pair efficiently. When the top predicted allele had at least 50% higher Pattern Score than the second predicted allele (Groups 2 and 4), median Top MH Fractions trended higher compared to Group 1 and 3, respectively. P-values calculated by Wilcoxon’s Each Pair Calculation (adjusted for multiple comparisons). Targets with < 20% overall edit efficiency were excluded from analysis. Pattern Scores were derived using RGEN online tool (http://www.rgenome.net). (TIF) [file pgen.1007652.s004.tif]

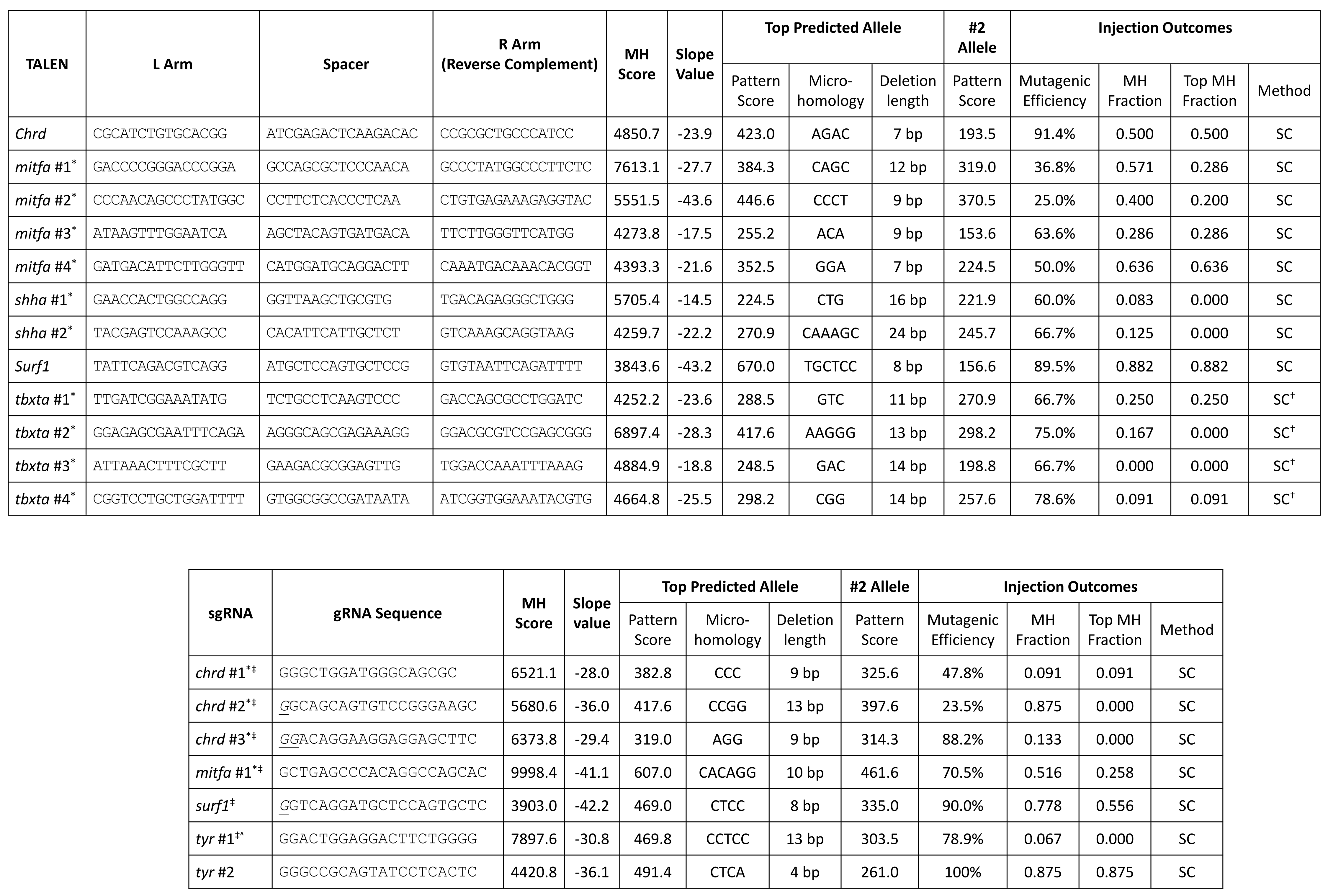

Supplement: S1 Table — Underlined & italicized bases in sgRNA sequence denote mismatched bases due to the promoter requirement. Pattern Scores and Microhomology Scores were derived using RGEN online tool (http://www.rgenome.net). MH: Microhomology, SC: Subcloning. * Reagents prospectively designed according to Bae, et al algorithm [14]. † No raw sequencing data were available. However, the outcome had been compiled into a table prior to conception of this study. ‡ Injected with sgRNA and Cas9 mRNA (150 pg and 100 pg, respectively). ^ Gift from Wenbiao Chen (addene # 46761). (TIF) [file pgen.1007652.s005.tif]

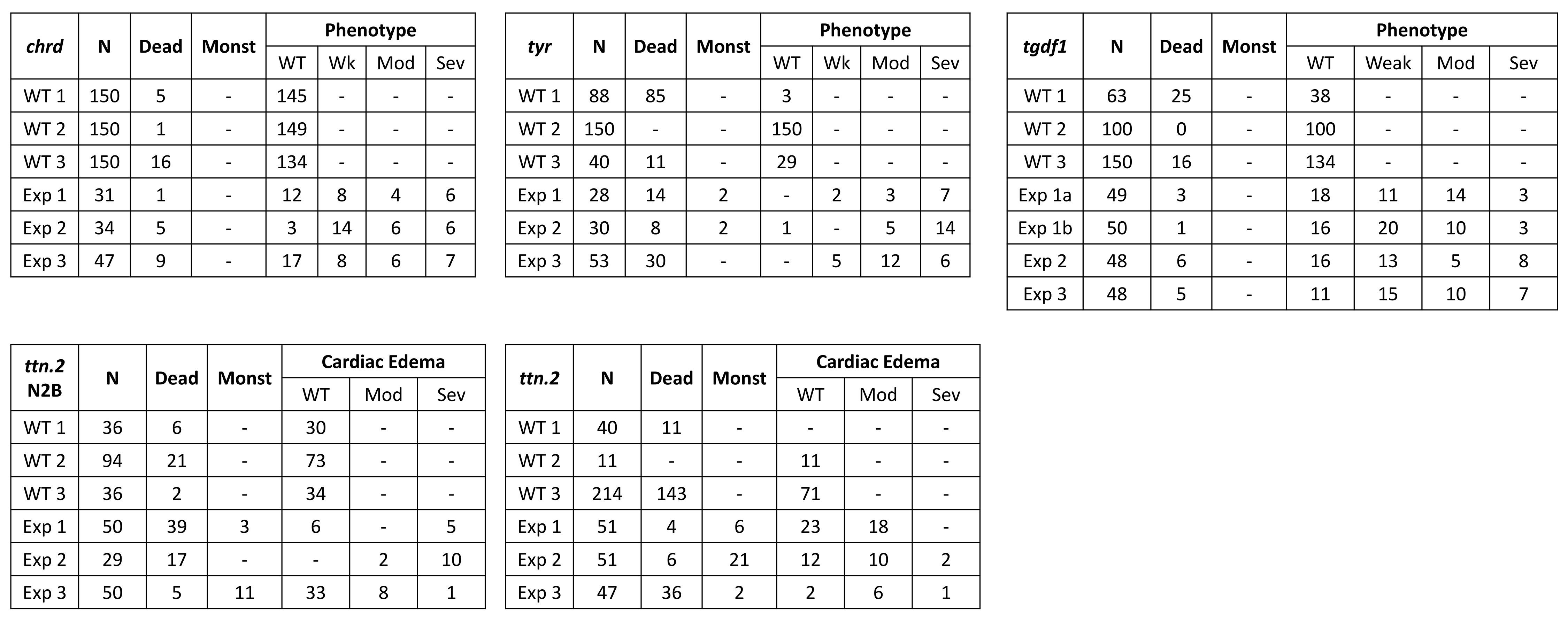

Supplement: S2 Table — For tdgf1, Experiments 1a and 1b correspond to technical replicates using WT 1 as reference, uninjected control. chrd and tdgf1 phenotypes were scored on 1 dpf, whereas tyr, ttn.2 N2B, ttn.2 phenotypes were scored on 2 dpf. (TIF) [file pgen.1007652.s006.tif]

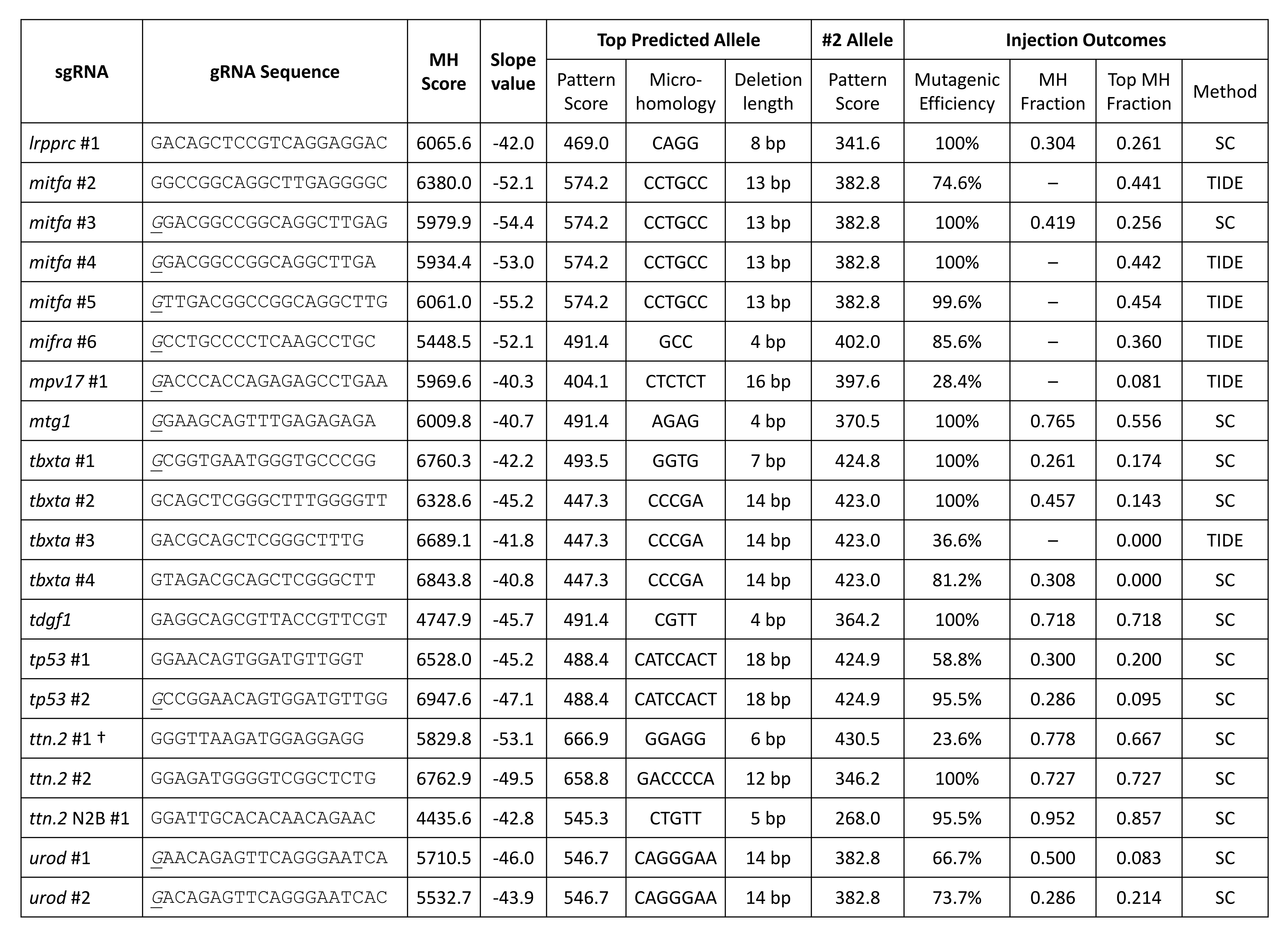

Supplement: S3 Table — Underlined & Italicized bases in gRNA sequence denote mismatched bases due to the promoter requirement. Pattern Scores and Microhomology Scores were derived using RGEN online tool. MH: Microhomology, SC: Subcloning, TIDE: Tracking Indels by DEcomposition. † Injected RNP at the dose of 115 pg sgRNA and 245 pg Cas9 due to poor viability at higher doses. (TIF) [file pgen.1007652.s007.tif]

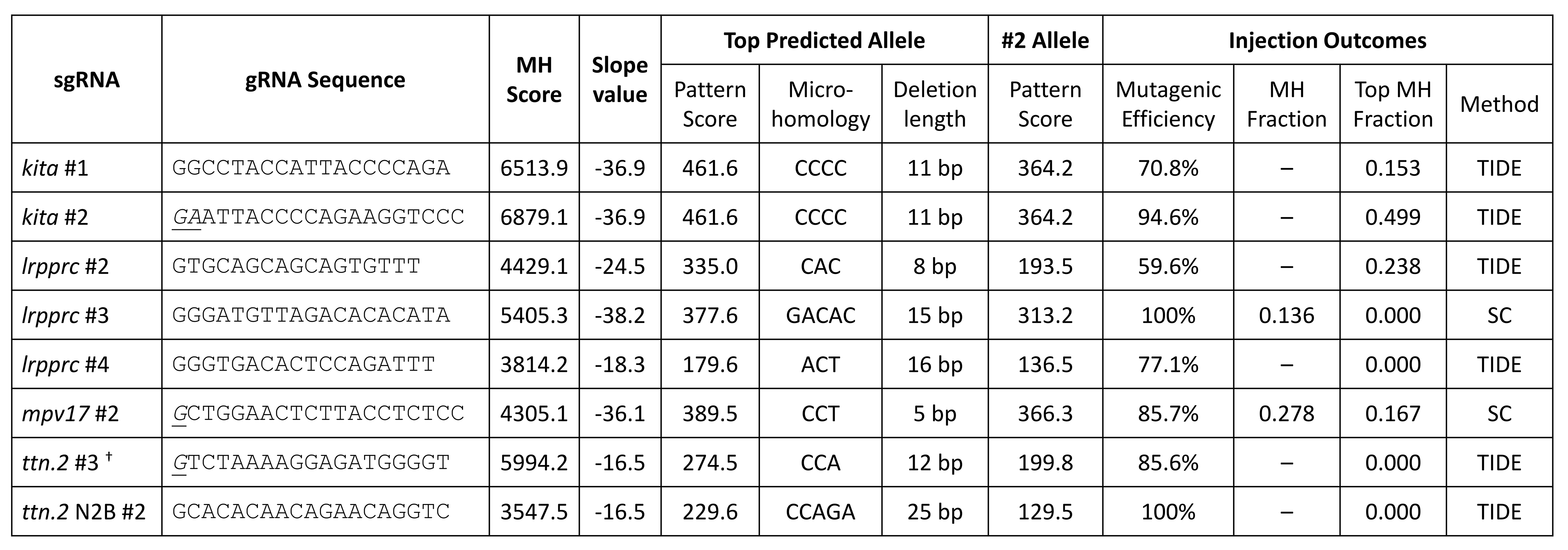

Supplement: S4 Table — Underlined & Italicized bases in sgRNA sequence denote mismatched bases due to the promoter requirement. Pattern Scores and Microhomology Scores were derived using RGEN online tool (http://www.rgenome.net). MH: Microhomology, SC: Subcloning, TIDE: Tracking Indels by Decomposition. † Injected RNP at the dose of 115 pg sgRNA and 245 pg Cas9 due to poor viability at higher doses. (TIF) [file pgen.1007652.s008.tif]

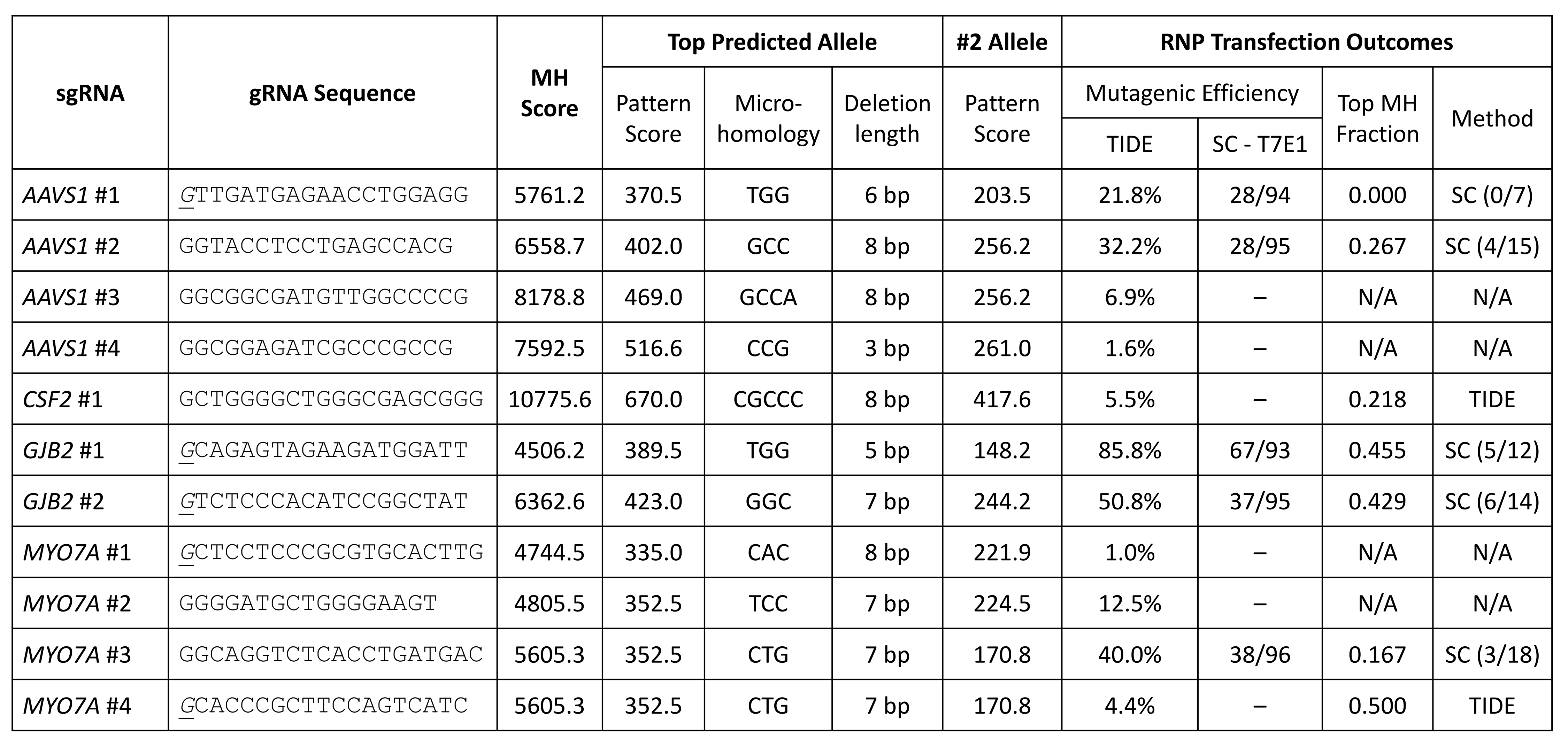

Supplement: S5 Table — Underlined & Italicized bases in sgRNA sequence denote mismatched bases due to the T7 promoter requirement. For loci wherein mutagenic efficiency and/or Top MH Fraction was calculated based on subcloning results, number of mutant/top predicted allele colonies are given in numerator and the total number of colonies analyzed are given in the denominator. Pattern Scores and Microhomology Scores were derived using RGEN online tool (http://www.rgenome.net). (TIF) [file pgen.1007652.s009.tif]

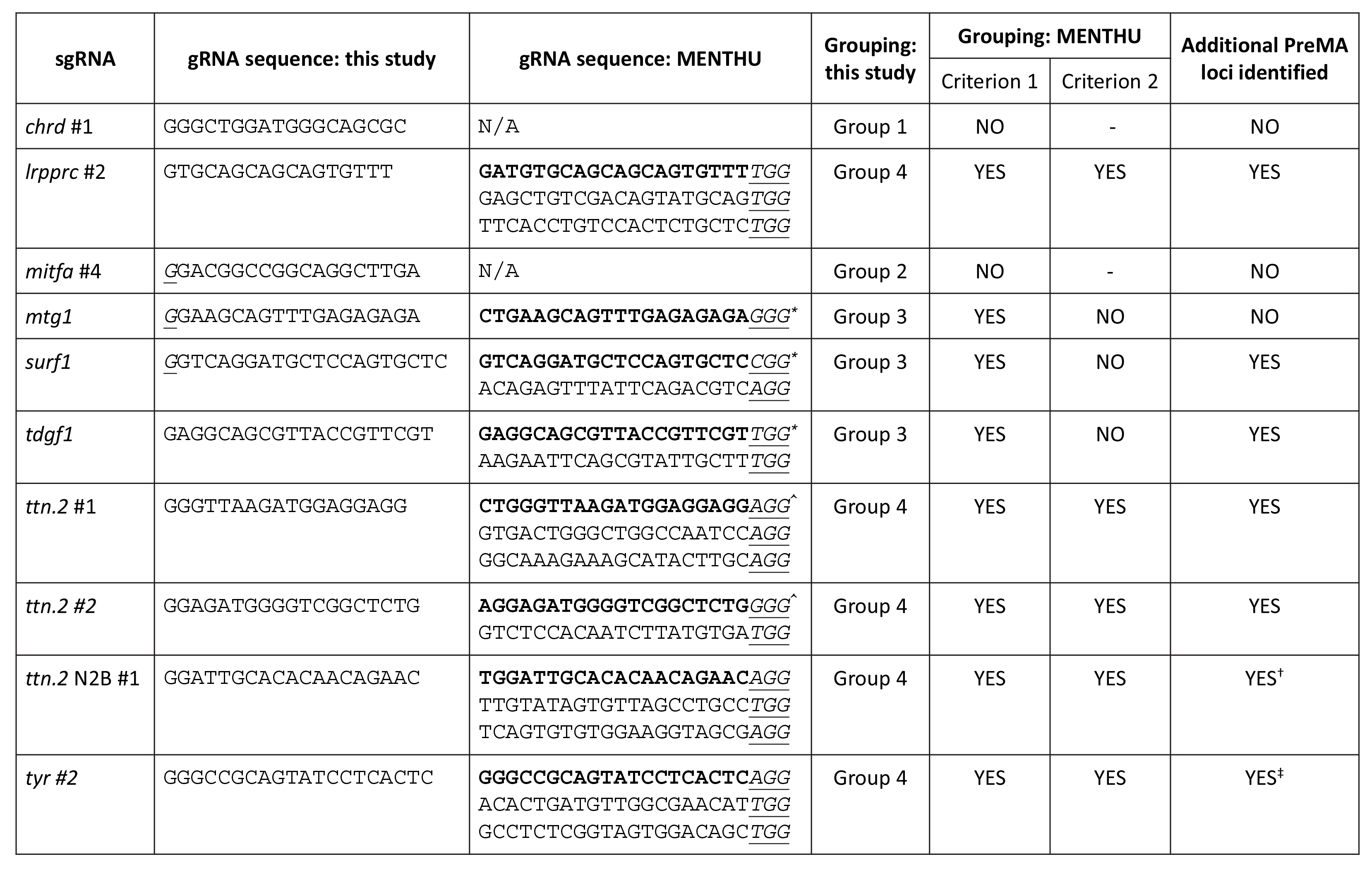

Supplement: S6 Table — The output was obtained by using the entire target exon sequence with 40 bp intronic sequence each on both 5’ and 3’ ends. The MENTHU output provides a 3’ NGG PAM sequence for each gRNA targets (italicized and underlined). MENTHU gRNA outputs that matched the target sequences used in this study are bolded. Criteria 1 and 2 refer to 1) if top predicted microhomology arm is separated by 5 bp or less, and 2) if the ratio of top to second predicted Pattern Scores is at least 1.5. MENTHU is programmed to terminate calculations if the target site is negative for Criterion 1. As a result, no gRNA sequence output is obtained for chrd #1 and mitfa #2. Importantly, in two instances (surf1 and tgdf1) where we only had Group 3 reagents, novel candidate PreMA sites were identified. * Result obtained by adjusting the value for Criterion 2 to 1.0 as these were Group 3 guides that, by definition, does not satisfy Criterion 2 of 1.5 or higher. ^ in-frame mutation by the experimental design. † 16 other candidate loci identified on this 3771 bp exon; only a partial list for alternate loci is given. ‡ 16 other candidate loci identified on this 822 bp exon; only a partial list for alternate loci is given. (TIF) [file pgen.1007652.s010.tif]

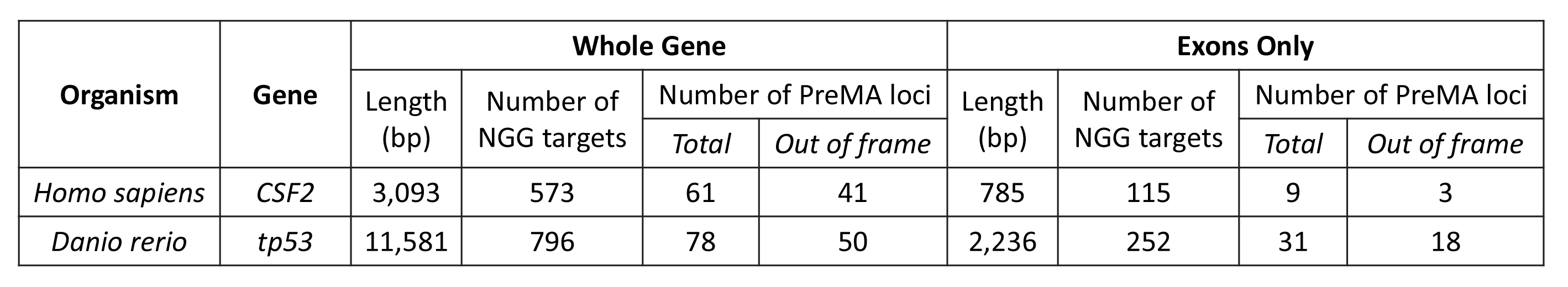

Supplement: S7 Table — This holds true for both at the gene and exonic levels. As expected, roughly two thirds of the PreMA reagents are predicted to induce preferentially out-of-frame mutations. (TIF) [file pgen.1007652.s011.tif]

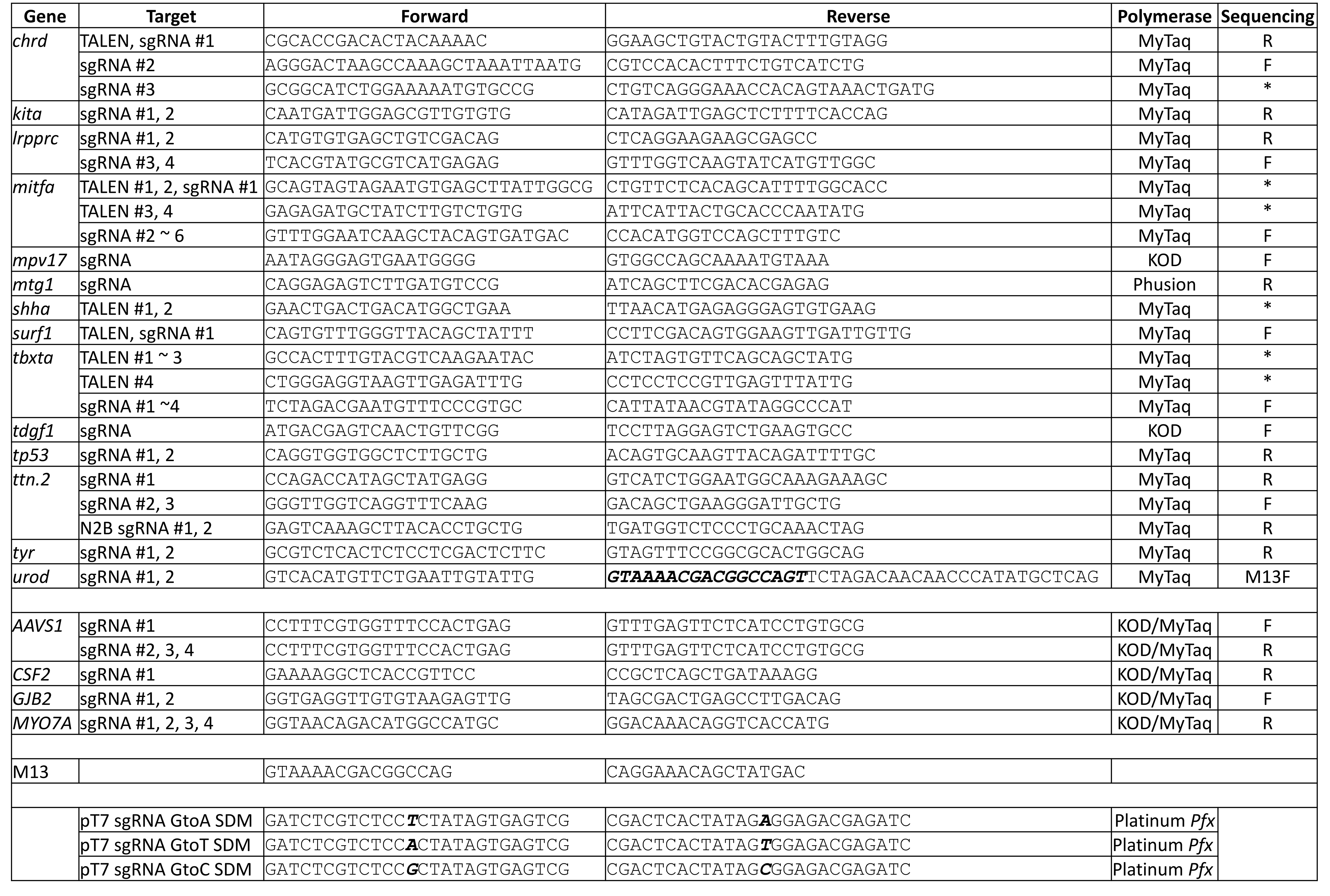

Supplement: S8 Table — All the primer sequences are provided in 5’ -> 3’ order. For urod Reverse primer, M13F primer sequence was added at the 5’ end of the endogenous target sequence (bolded and italicized). For SDM primers, intended point mutation is indicated by bold and italic. * No endogenous primer was used to sequence the genomic loci of interest. (TIF) [file pgen.1007652.s012.tif]
