# Supplementary material for: Robust activation of microhomology-mediated end joining for precision gene editing applications
Source: PLoS Genet. 2018 Sep 12;14(9):e1007652. doi: 10.1371/journal.pgen.1007652 (PMC6152997; doi:10.1371/journal.pgen.1007652)
Supplement: S1 Note — (DOCX) [file pgen.1007652.s013.docx]

**S1 Note** Calculation of Microhomology Fraction

1. When the mutagenic outcomes were assessed by subcloning, the Microhomoloy Fraction was calculated according to the formula below:

$$MH Fraction= \frac{(\# of mutant colonies harboring 3bp microhomology or longer)}{(\# of total mutant colonies recovered)}$$

2. When the mutagenic outcomes were assessed by TIDE analysis, the Microhomology Fraction was not calculated
3. For HeLa cell data, Microhomology Fraction was calculated as below, discarding any alleles with allele frequency of < 0.1%.

$$MH Fraction= \frac{(\# of mutant reads harboring 3bp microhomology or longer)}{(\# of total mutant reads recovered)}$$
