# Supplementary material for: Robust activation of microhomology-mediated end joining for precision gene editing applications
Source: PLoS Genet. 2018 Sep 12;14(9):e1007652. doi: 10.1371/journal.pgen.1007652 (PMC6152997; doi:10.1371/journal.pgen.1007652)
Supplement: S3 Note — (DOCX) [file pgen.1007652.s015.docx]

**S3 Note** Calculation of Top Microhomology Fraction

1. When the mutagenic outcomes were assessed by subcloning, the Top Microhomoloy Fraction was calculated according to the formula below:
2. When the mutagenic outcomes were assessed by TIDE analysis, the Top Microhomology Fraction was calculated according to the formula below:

$$Top MH Fraction= \frac{\begin{aligned} (\% mutat population corresponding with the same number of bases \\ deleted as top predicted allele with 3bp microhomology or longer) \end{aligned}}{(100\% - \% 0bp change population)}$$

e.g) *ttna* sgRNA #1 (**See below for TIDE output**)


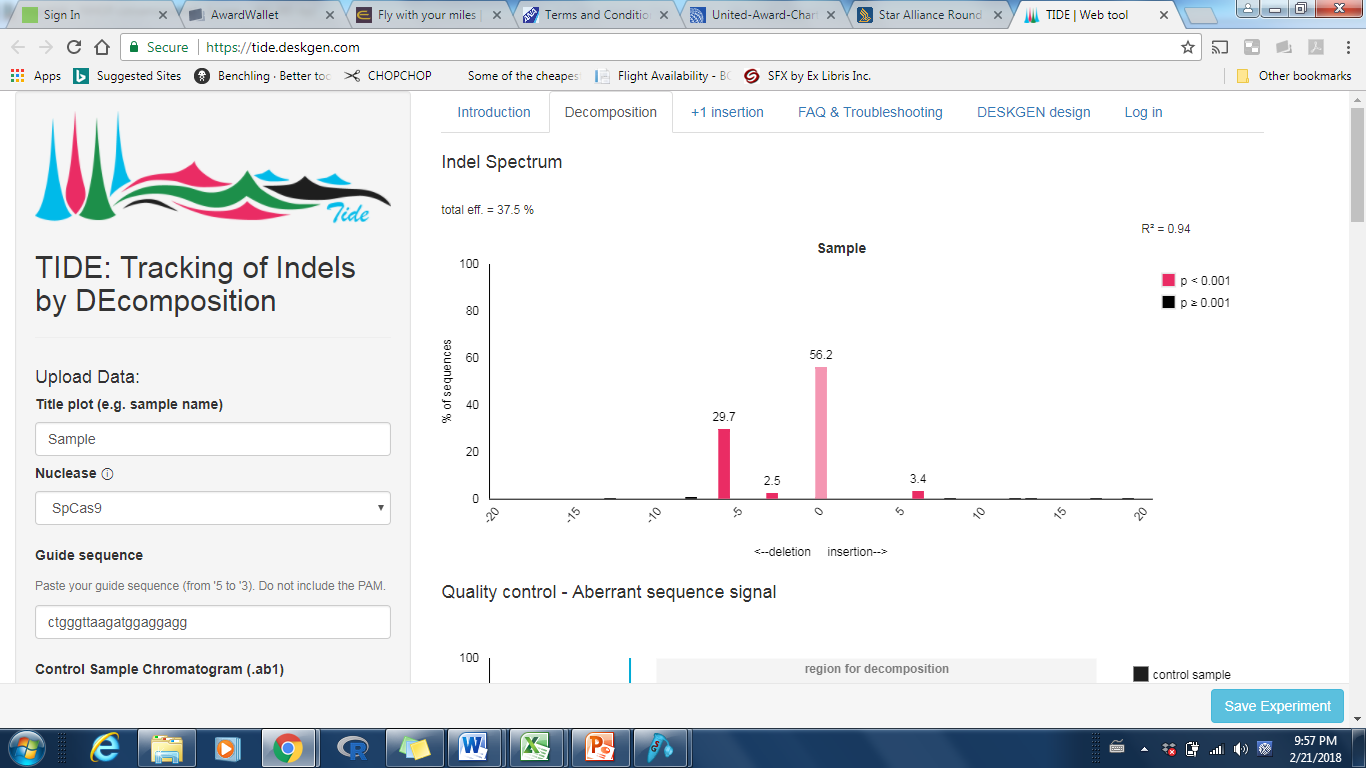

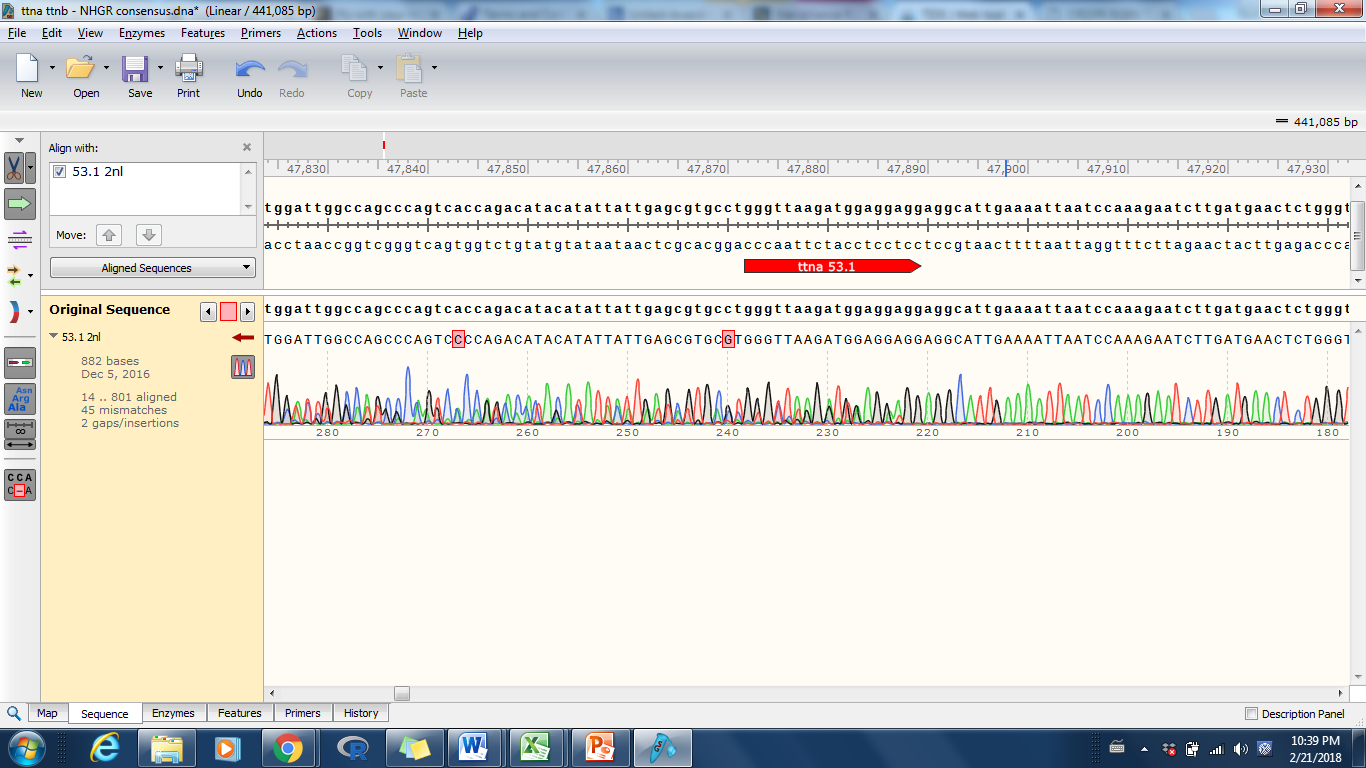

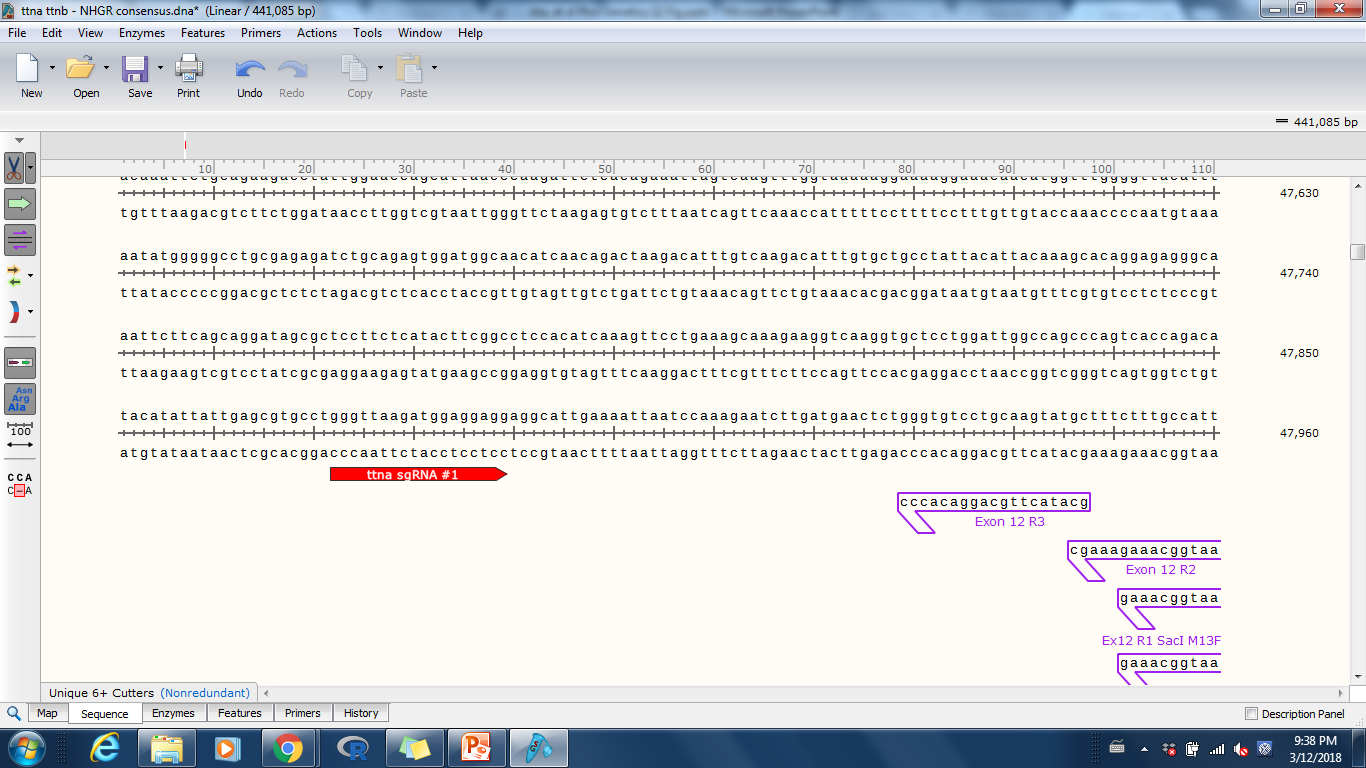


1. For HeLa cell data, Microhomology Fraction was calculated as below, discarding any alleles with allele frequency of < 0.1%.
