# Supplementary material for: Robust activation of microhomology-mediated end joining for precision gene editing applications
Source: PLoS Genet. 2018 Sep 12;14(9):e1007652. doi: 10.1371/journal.pgen.1007652 (PMC6152997; doi:10.1371/journal.pgen.1007652)
Supplement: S1 Data — Whole amplicon sequencing outcomes are deposited as .ab1 files for chrd TALEN and tyr #2, tdgf1, ttn.2 N2B #1, ttn.2 #2 sgRNA targets. Other sequencing outcomes, including those used for subcloning analyses are provided in the .fastq formats. (ZIP) [file pgen.1007652.s016.zip › Ata,_et_al_Plos_Genetics_Supplemental_Notes.pdf]

S1 Note Calculation of Microhomology Fraction

1) When the mutagenic outcomes were assessed by subcloning, the Microhomoloy Fraction was calculated according to the formula below:

$$\text{MH Fraction} = \frac{(\# \ of \ mutant \ colonies \ harboring \ 3bp \ microhomology \ or \ longer)}{(\# \ of \ total \ mutant \ colonies \ recovered)}$$

2) When the mutagenic outcomes were assessed by TIDE analysis, the Microhomology Fraction was not calculated
3) For HeLa cell data, Microhomology Fraction was calculated as below, discarding any alleles with allele frequency of < 0.1%.

$$\text{MH Fraction} = \frac{(\# \ of \ mutant \ reads \ harboring \ 3bp \ microhomology \ or \ longer)}{(\# \ of \ total \ mutant \ reads \ recovered)}$$

S2 Note Calculation of Slope Values

The Slope Values were calculated for each target locus as follows:

1) Input 80bp endogenous gene sequence flanking the predicted DSB site into the Microhomology-Predictor (http://www.rgenome.net/mich-calculator/){Bae, 2014 #1}.
    a. In the case of CRISPR-Cas9 reagents, phosphodiester bond between the 3rd and 4th base pairs distal to the PAM was chosen as the presumptive DSB site. Subsequently, 40bp each on both sides of this break site was used as the input sequence.
    b. In the case of TALEN reagents with even number of bases in the Spacer region, the phosphodiester bond between the 5' and 3' halves of the Spacer was chosen as the presumptive DSB site. Subsequently, 40bp each on both sides of this break site was used as the input sequence.
    c. In the case of TALEN reagents with odd number of bases in the Spacer region, the center-most base that bridges 5' and 3' halves was identified. Subsequently, a 79bp sequence containing this center-most base and 39bp each on both sides of this base was used as the input sequence.
2) Ranked the top 10 candidates by the Pattern Score in a descending order.
    a. In the dataset wherein only microhomology arms of 3bp or greater were considered, candidate mutant alleles harboring only 2bp microhomology arms were omitted from further analysis.
    b. In the dataset wherein microhomology arms of 2bp or greater were considered, no candidate mutant alleles were omitted.
3) Plotted the Pattern Scores against the numerical rank on Scatter Plot using Microsoft Excel
    a. Drew simple linear regression
    b. The "a" in the fitted line formula $y = a * x + b$ is the Slope Value
4) The steeper (i.e. larger absolute value for a) the slope, the lower influence there is from the competing locally available microhomology arms. For zebrafish, the cutoff used for Low Competition sites was -40.
5) The flatter (i.e. values closer to 0 for a) the slope, the more influence there is from the competing locally available microhomology arms. For zebrafish, the cutoff used for High Competition sites was -20.

S3 Note Calculation of Top Microhomology Fraction

1) When the mutagenic outcomes were assessed by subcloning, the Top Microhomoloy Fraction was calculated according to the formula below:

$$\text{Top MH Fraction} = \frac{(\qquad of\ colonies\ with\ mutation\ corresponding\ to\ top\ predicted\ allele\ with\ 3bp\ microhomology\ or\ longer)}{(\#\ of\ total\ mutant\ colonies\ recovered)}$$

2) When the mutagenic outcomes were assessed by TIDE analysis, the Top Microhomology Fraction was calculated according to the formula below:

$$\text{Top MH Fraction} = \frac{(\%\ mutat\ population\ corresponding\ with\ the\ same\ number\ of\ bases\ deleted\ as\ top\ predicted\ allele\ with\ 3bp\ microhomology\ or\ longer)}{(100\% - \%\ 0bp\ change\ population)}$$

e.g) *ttna* sgRNA #1 (**See below for TIDE output**)

$$\text{Top MH Fraction} = \frac{29.7\%}{(100\% - 56.2\%)}$$

$$= 0.678$$

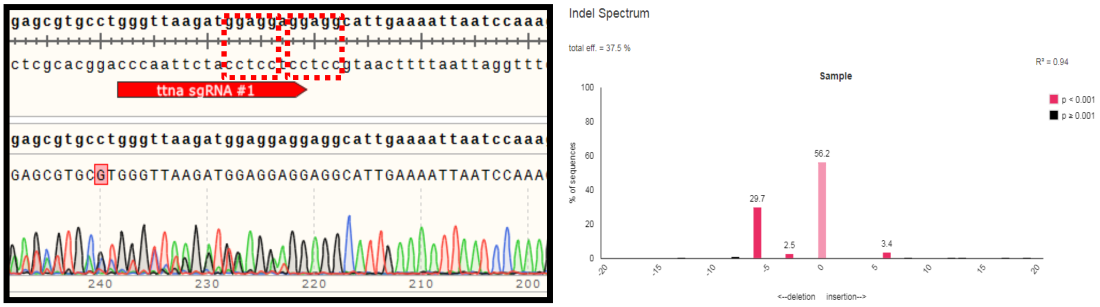

3) For HeLa cell data, Microhomology Fraction was calculated as below, discarding any alleles with allele frequency of < 0.1%.

$$\text{Top MH Fraction} = \frac{(\qquad of\ reads\ with\ mutation\ corresponding\ to\ top\ predicted\ allele\ with\ 3bp\ microhomology\ or\ longer)}{(\#\ of\ total\ mutant\ reads\ recovered)}$$
